# Loss-of-function mutations are main drivers of adaptations during short-term evolution

**DOI:** 10.1038/s41598-024-57694-8

**Published:** 2024-03-26

**Authors:** Joanna Klim, Urszula Zielenkiewicz, Szymon Kaczanowski

**Affiliations:** Institute of Biochemistry and Biophysics, Polish Academy of Sciences, Pawinskiego 5a, 02-106 Warsaw, Poland

**Keywords:** Genome evolution, Molecular evolution, Population genetics

## Abstract

We noticed that during short-term experimental evolution and carcinogenesis, mutations causing gene inactivation (i.e., nonsense mutations or frameshifts) are frequent. Our meta-analysis of 65 experiments using modified dN/dS statistics indicated that nonsense mutations are adaptive in different experimental conditions and we empirically confirmed this prediction. Using yeast *S. cerevisiae* as a model we show that fixed or highly frequent gene loss-of-function mutations are almost exclusively adaptive in the majority of experiments.

## Introduction

Recent progress in genomics has revealed that gene loss is a pervasive source of genetic variation. Nevertheless, it is unclear whether most gene loss-of-function mutation fixations are neutral or adaptive^1^. An alternative and not fully exclusive possible explanation for evolution by gene loss is “regressive evolution”^1^, also named “trait/DNA decay”^2,3^. According to this hypothesis, when a trait is no longer under stabilizing selection in given conditions, mutations accumulate in genes that are associated with that trait. As a result, due to rather neutral evolution, organisms may show a loss of adaptations that may be required in other conditions. Thus, the main goal of this study is to experimentally test the classical hypothesis that claims that loss-of-function mutations play a basic role in rapid adaptation to ecological conditions (i.e., that observed loss-of-function mutations are beneficial).

Classical studies on the pathological evolution of cancer cells have revealed that neoplastic transformation is driven by loss-of-function mutations in cancer suppressors^4^. In cancer cells, complex traits that have evolved over millions of years are inactivated. Malignant transformation can be seen as a reversion from the phenotype of a differentiated cell of a multicellular organism to an ancestral unicellular eukaryotic phenotype^5^. During neoplastic transition, malignant cells typically “switch” from mitochondrial aerobic to cytoplasmic anaerobic respiration (Warburg’s hypothesis of cancer origin)^6^. Therefore, cancer cells lose various traits involved in mitochondrial domestication. In one of our reports, we suggested that apoptosis is involved in the Warburg effect^7^.

The rapid generation of putative loss-of-function mutations has also been observed in various experimental studies involving microorganisms^8^. The most extreme cases of genome reduction have been observed in various microorganisms, including endosymbiotic bacteria^9^.

There is substantial experimental evidence that loss-of-function mutations can be adaptive. It has been shown that loss-of-function mutations enhance the rate of adaptation and evolvability, which means they can be considered as a gateway to evolutionary innovation and rapid adaptation^10^. The observable high rate of loss-of-function mutations occurrence points that these mutations occupy a prominent place in the adaptation of bacterial populations to new environments and can provide significant fitness advantages under various challenging conditions. Hottes et al.^11^ analysed genome-wide fitness data across 144 conditions and they noticed that adaptive null mutations are highly abundant. Lang and co-workers^12^ followed the evolution of *S. cerevisiae* in rich medium for 1000 generations. They found that 69 (of a total 141) of putative adaptive mutations were either frameshift or nonsense mutations, which one can expect to result in a loss of gene function. Recently, Monroe and colleagues reviewed a number of classical studies documenting adaptive loss-of-function events^13^. However, as mentioned before, in light of current knowledge, it is unclear whether most gene loss fixation is neutral or adaptive. As pointed out by Albatal and Cañestro^1^, clarifying this issue will constitute an important contribution to the wider and still open neutralism-selectionism debate on whether genetic variation in populations is mostly neutral or adaptive and whether neutral variants are relevant to the emergence of evolutionary innovations.

Here, we tested the hypothesis that mutations leading to gene inactivations are beneficial using the short-term experimental evolution of yeast *S. cerevisiae.* Short-term experimental evolution is a classical method of mutant selection that has gained much interest mainly due to the development of high-throughput sequencing. The main challenge of this methodology is predicting, which mutations are “significant” drivers of adaptations and which are merely passengers. The standard approach includes parallel evolutionary experiments, whole genome sequencing, mutation detection, and experimental confirmation. We recently suggested that in the case of gene loss-of-function mutations, a simplified approach could be applied using a *dN*/*dS* test only for nonsense mutations^14^. This method reveals whether mutations leading to gene inactivation tend to be beneficial. Briefly, the dN/dS method assumes that the probability of mutation is equal in silent and non-silent sites of a given gene. However, natural selection has an impact on the probability of fixing and spreading a given mutation in the population. The *dN*/*dS* method quantifies selection pressures by comparing the rate of substitutions at silent sites (*dS*), which are presumed neutral, to the rate of substitutions at non-silent sites (*dN*). When a rapid change of protein sequence is evolutionarily beneficial, nonsynonymous mutations are observed more frequently than synonymous ones, i.e., *dN*/*dS* > 1 and *dN* > *dS*. The observed surplus of nonsynonymous mutations (*dN*-*dS*) is fixed due to positive selection. Conversely, when a change in the protein sequence is deleterious, it is frequently removed and then *dN/*dS < 1 and *dN* < *dS*. The observed deficit of non-synonymous mutations (*dS*-*dN*) is caused by purifying selection which removes deleterious mutations.

In our previous study, we demonstrated evidence that nonsense mutations are drivers of adaptations during the compensatory evolution following gene deletion^14^. There, instead of nonsynonymous substitution rates (dN), we calculated missense (*dMISSENSE*) and nonsense (*dNONSENSE*) substitution rates for the entire genomes. Here, we applied this methodology to investigate the mechanism of adaptations of the model organism yeast *S. cerevisiae*.

## Results and discussion

**Figure 1:**
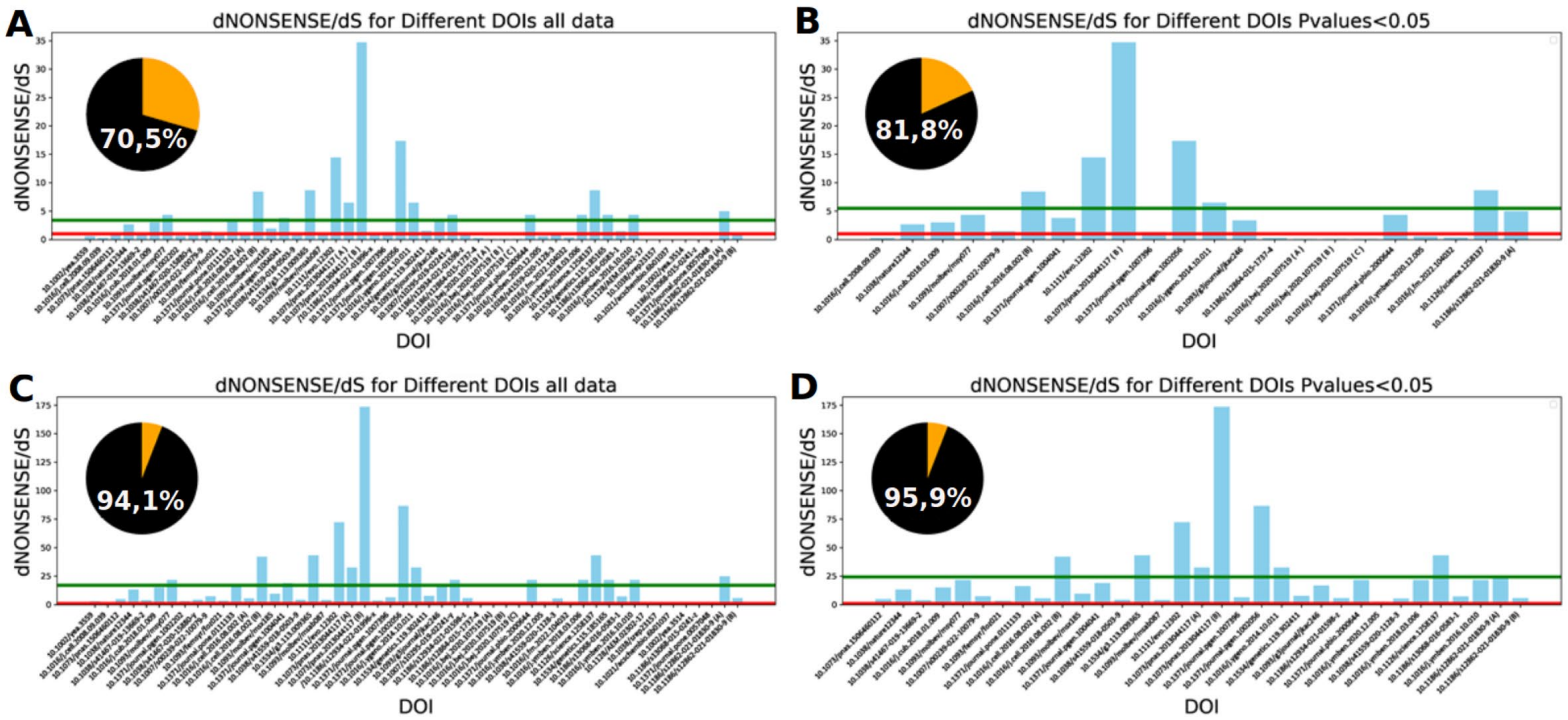
The estimations of fraction of beneficial nonsense mutations in various studies.

In this study, we checked whether it applies as a general rule that gene loss-of-function mutations fixed during short-term evolution are beneficial drivers of adaptations. To answer this question, we performed a meta-analysis of **58** published studies providing information about **65** evolutionary experiments which included whole genome sequencing data (detailed calculations and applied computer programs are shown in Python Jupyter notebook (SI Dnonsense_calculations.pdf) and obtained results are presented in an Excel spreadsheets (SI Dnonsense_calculations.xlsx). In the case of **55** experiments, data contained information about synonymous mutations and were considered in the meta-analysis. In 17 cases, the nonsense substitution rate was significantly higher than the synonymous substitution rate (*dNONSENSE* > *dS*, *p*-value < 0.05), in 7 cases, it was lower (*dNONSENSE* < *dS*), and in 31, it was statistically equal (Fig. 1A,B). To sum up, the rate of accumulation of nonsense mutations and synonymous mutations was approximately equal during short-term evolution (*dNONSENSE* $\simeq$ *dS*). However, as genes are generally evolutionarily conserved, disruption of the majority of genes will likely be deleterious. As a result, only a small fraction of gene loss-of-function mutations will be beneficial during short-term evolution. This assumption was confirmed by different experimental studies^15–17^. For example, a quite recent controversial experimental study confirmed this expectation^17^. It demonstrated that nonsense mutations of non-essential genes are non-lethal deleterious, while it was unclear whether synonymous mutations are neutral or very weakly deleterious.

We estimated the fraction of nonsense deleterious mutations using *dN/dS* (*dNonsense/dS*) statistics. In case when the ratio is smaller than 1 it shows what is a fraction of non-synonymous mutations removed by purifying selection. Therefore, we attempted to estimate the fraction of nonsense mutations removed by selection. The comparisons between different yeast laboratory strains suggested that ~ 98% of nonsense mutations are deleterious and removed by purifying selection in the natural environmental conditions (*dNONSENSE*/*dS* $\simeq$ 0.02). We also determined that the lowest ratio for *dNONSENSE*/*dS* (0.09–0.1) is noted in the case of three data sets obtained during experimental evolution combined with mutagenesis^15^. We supposed that mutagenesis causes mutational load. In conclusion, experimental data indicated that the purifying selection removes even 90% of appearing lethal nonsense mutations. Assuming that 80–90% of nonsense mutations are lethal and removed by the purifying selection, and other nonsense mutations are neutral or deleterious, the rate at which nonsense mutations accumulate should be at least five times slower than for synonymous mutations *(dNONSENSE* < 0.2 × dS). In such a case, the surplus of nonsense mutations (*dNONSENSE*-0.2 × dS) will be driven by positive selection (Fig. 1C,D). To sum up, our data analysis indicates that the elevated frequency of nonsense mutations that lead to gene inactivation, observed during short-term evolution (Fig. 1), is caused by their selective advantage (i.e., beneficial effect). Similar conclusions were drawn by the Campbell group regarding the pathological evolution of cancer cells^18^.

The figure illustrates the *dNONSENSE*/*dS* ratio with an estimated fraction of beneficial nonsense mutations (inset plot), calculated as (mean(*dNONSENSE*/*dS*)-1)/mean(d*NONSENSE*/*dS*). In the plot, the green line represents the mean *dNONSENSE*, while the red line corresponds to the value of 1. (A) The analysis for all studies with the assumption that all nonsense mutations/gene inactivations are neutral. (B) The same analysis as in (A), but limited to studies where *dNONSENSE* and dS were statistically different. (C) The analysis for the assumption that 80% of nonsense mutations are lethal and thus excluded from calculations of *dNONSENSE*, leaving only 20% considered neutral. (D) The same analysis as in (C), but limited to studies where *dNONSENSE* and *dS* were statistically different.

Studies where *dS* = 0 were excluded due to the difficulty in estimating the *dNONSENSE/dS* ratio, as it is likely very high.

To support this conclusion, we performed another meta-analysis. We assumed that there is a higher probability that a gene will be inactivated by nonsense mutation than by missense mutation. Following this expectation, the positive selection acting on gene loss-of-function mutations would lead to a higher nonsense mutation rate than missense mutation rates. Our analyses showed that the nonsense mutation rate was higher than the missense mutation rate in the majority (74%) of cases of short-term experimental evolution. In contrast, a comparison of yeast lab isolates revealed that the nonsense mutation rate is about 10 times lower than the missense mutation rate (SI Dnonsense_calculations.xlsx). In contrast to the experimental evolution in novel ecological conditions, these lab populations evolve in a relatively constant environment. This observation shows that there are very strong selection constraints preserving gene content.

In conclusion, our theoretical predictions indicate that gene loss-of-function mutations that are not eliminated by purifying selection are drivers of adaptations in short-term evolution. However, it is not clear to what extent the *dN/dS* approach may predict the impact of mutations in evolutionary experiments. This method is based on the controversial assumption that synonymous mutations are generally neutral. Moreover, the already mentioned recent study by Zhang and co-workers showed that three-quarters of synonymous mutations resulted in a significant reduction in fitness, and the distribution of fitness effects was overall similar between synonymous and nonsynonymous mutations^17^.

Therefore, we experimentally tested the hypothesis that gene loss-of-function mutations are drivers of adaptations. For this purpose, we used our genomic data obtained during short-term evolutionary experiments of yeast *S. cerevisiae* in continuous culture^19^. By conducting experimental evolution followed by whole genome sequencing of various mutator and non-mutator yeast populations, we identified a set of polymorphic mutations (with a 20% mutation frequency threshold). In both cases, nonsense mutation rates were higher than synonymous ones (dNonsense/dS = 4.96 for non-mutator strains and 1.14 for mutator strains), indicating positive selection. Likely, a higher mutational load causes an increased frequency of deleterious nonsense mutations in mutator strains. According to this, after the initial phase of rapid accumulation of advantageous gene loss-of-function mutations, subsequent nonsense mutations tend to be exclusively deleterious. However, purifying selection removes 80% of nonsense mutations, and all observed are beneficial. As a result, the dNONSENSE/dS ratio is about five times higher in non-mutator strains than in mutator ones.

**Figure 2:**
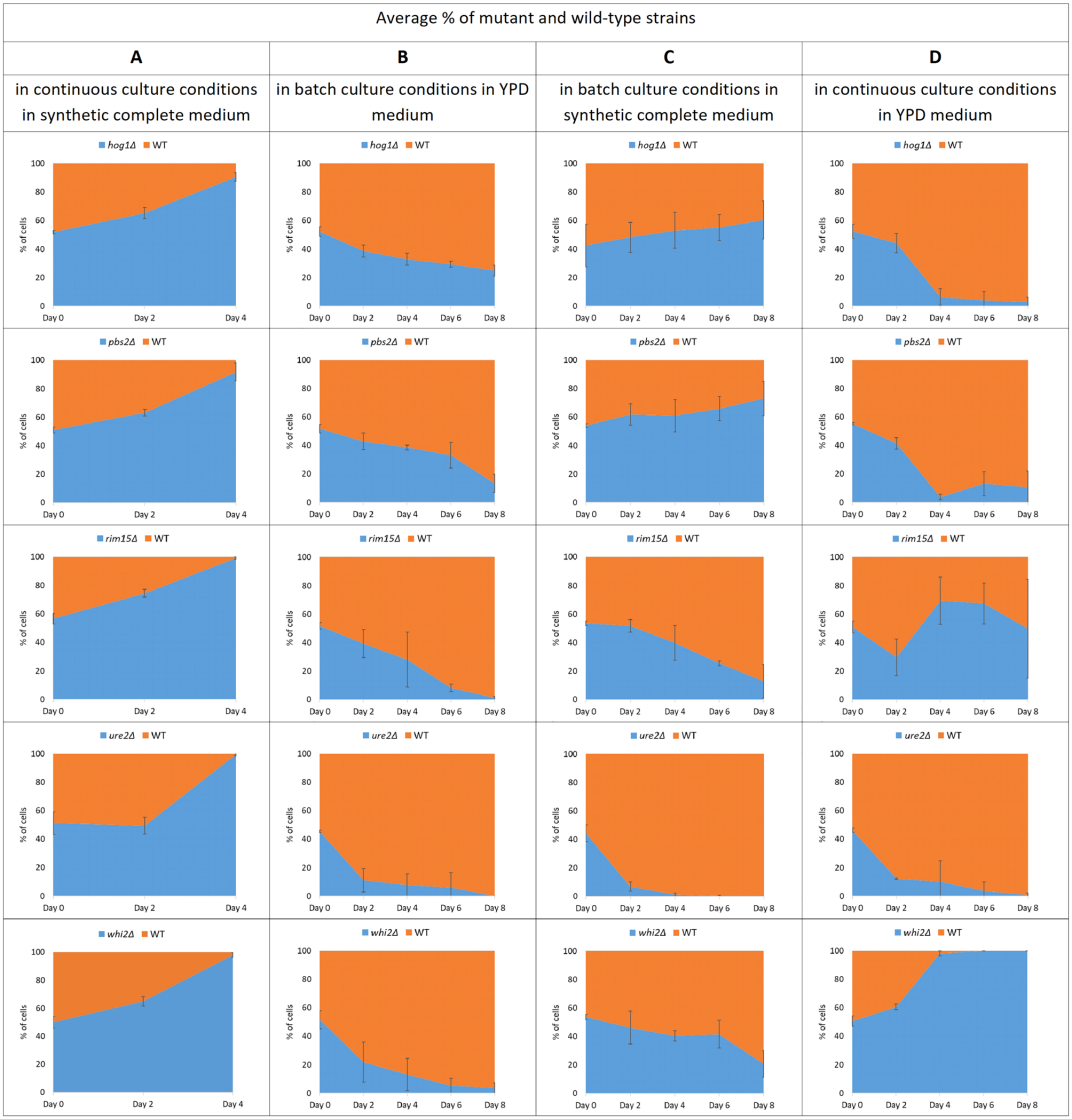
Competition of wild-type and mutant *S. cerevisiae* strains under various culturing conditions. Competition assays were performed for *hog1Δ, pbs2Δ*, *rim15Δ*, *ure2Δ*, *whi2Δ,* and wild-type *S. cerevisiae* W303 strains. The cell ratio of the mutant and wild-type strains was monitored by plating ca. 100 cells onto the YPD solid medium and replicating grown colonies onto the same medium supplemented with geneticin to distinguish the mutant cells from the wild-type cells. Data for each graph represents the mean ∓ SD of at least three independent assays.

We corroborated our conclusion by testing the proliferation rate (under various experimental conditions) of wild-type and mutant *S. cerevisiae* strains devoid of genes that gained loss-of-function mutations during our above-mentioned evolutionary experiments^19^. Results of our study suggested that gene loss-of-function mutations in five genes (*WHI2, URE2, RIM15, HOG1, PBS2*) are beneficial. In these genes, mutations were recurrent and some of them were nonsense or led to frameshifts (in the case of non-mutator strains the majority of nonsense and frameshift mutations occurred in these genes). It is worth mentioning that nonsense mutations usually activate a mechanism named nonsense-mediated RNA decay and have a similar impact to gene deletions^20^. To characterize the effects of deletion of the predicted genes on yeast growth and fitness, we constructed respective knockout strains and performed a set of competition experiments comparing the proliferation rates of wild-type and deletion mutant strains co-cultivated in various experimental conditions, i.e., in continuous and batch cultures using a complete synthetic liquid medium or YPD, respectively. Co-cultures were plated and the colonies of the wild-type and respective mutant strains were enumerated (Fig. 2). We found that the ancestor wild-type strain is losing to the competition with deletion strains when cultivated in the exact same growth conditions as these applied during experimental evolution (Fig. 2A) and outperforming most of the mutants when cultivated in the standard laboratory conditions (Fig. 2B) which suggests that the identified mutations could, in fact, be adaptive and selected in response to utilized experimental conditions.

For competition experiments, we also applied “mixed” conditions. Strains were co-cultivated in YPD medium as continuous cultures, and in complete synthetic medium in batch cultures. We observed differences in the final results for these two cultivating scenarios which were not identical even in terms of the same experimental set. When co-cultures were grown in a synthetic complete medium but were periodically refreshed to a new medium batch, the given mutant strain significantly outperformed the wild-type strain in only one in five cases (*pbs2Δ*) (Fig. 2C). Likewise, when co-cultures were grown in a YPD medium but in a continuous manner, mutant strain significantly outperformed the wild-type strain only one in five cases (*whi2Δ*) (Fig. 2D).

Interestingly, the *ure2Δ* mutant won the competition only when identical conditions (as these utilized in short-term evolutionary experiments) were applied which strongly suggests that potential loss-of-function mutations that appeared in the *URE2* gene during laboratory evolution are in fact adaptive.

The fact that yeast did not lose these genes during evolution indicates that existing purifying selection removes gene loss-of-function mutations of these genes, i.e., they have an adaptive function in natural ecological conditions. Our analysis of orthology revealed that such purifying selection is an evolutionary old process, as all these genes have orthologs in other fungal organisms and three in remotely related non-fungal eukaryotes (namely *URE2*, *RIM15*, and *HOG1*). On the other hand, only one of these genes (*URE2*) has a second ortholog in yeast, which could potentially substitute for the beneficial function of the deleted gene copy.

## Conclusions

The presented results confirm expectations of the classical “less is more” hypothesis that loss-of-function mutations play a basic role in rapid adaptation to ecological conditions. During this process, evolutionarily conserved genes are deleterious in new conditions^1,21^.

This observation is significant for the interpretation of results of evolutionary experiments. Using whole genome sequencing data, one can easily predict mutations causing gene loss-of-function such as nonsense, frameshift, or destabilizing protein structure. Likely, such mutations are almost exclusively beneficial.

The presented study shows that the *dN/dS* approach may predict the impact of mutations in evolutionary experiments.

## Methods

### Meta-analyses

**Figure 3:**
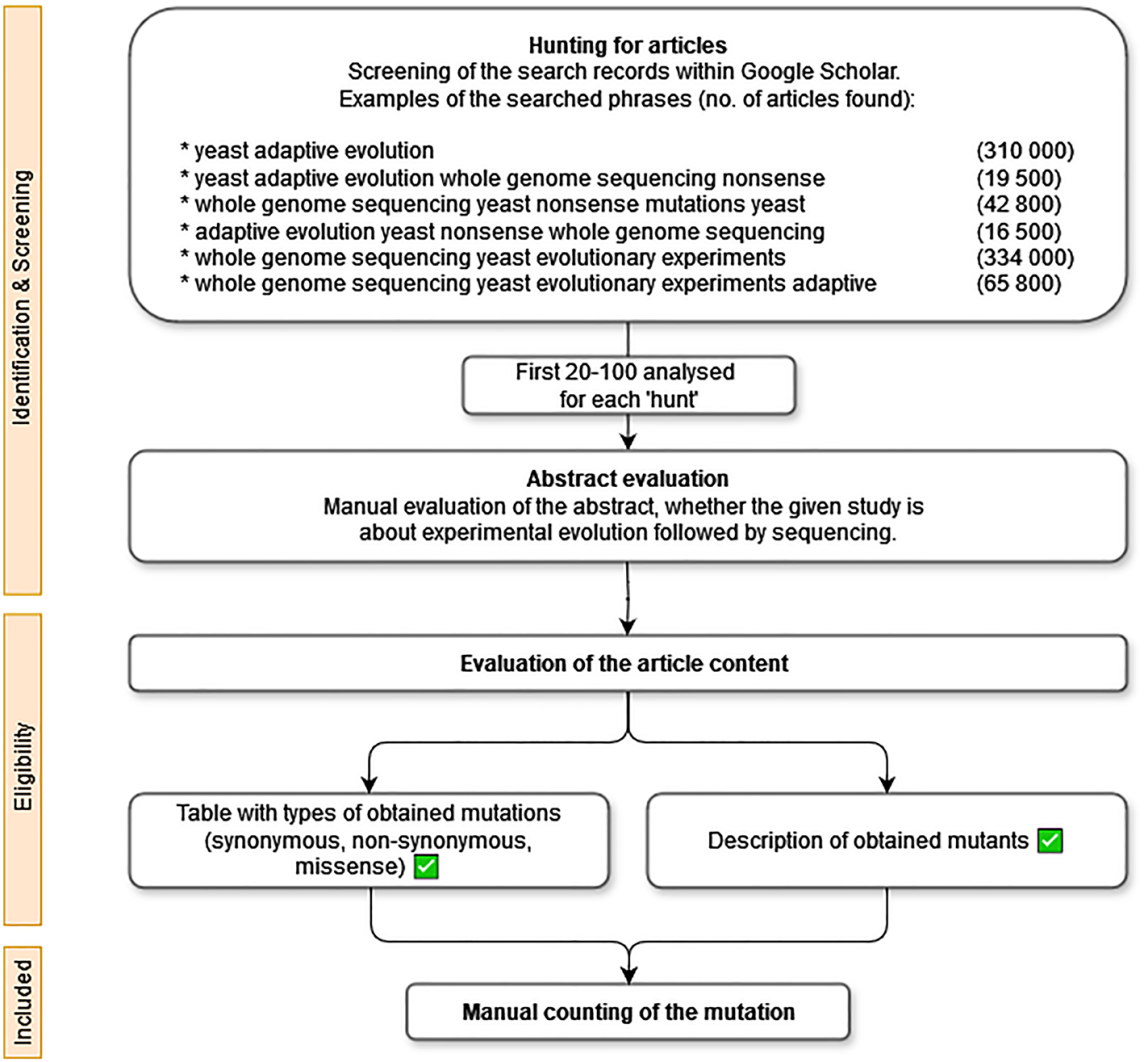
Flow chart showing articles and data selection process.

Data were obtained by conducting literature mining as shown in the PRISMA scheme (Fig. 3). Evolutionary experiments with haploid and diploid yeasts were included in the analysis. Evolutionary mutation rates (synonymous, missense, and nonsense) for the whole genome were calculated using a modified Miyata and Yasunaga method^14,22^, without correction for multiple substitutions at the same site. This correction is not needed as rates are very low.

The statistical significance was calculated by chi-square test.

Detailed calculations and applied computer programs are shown in Python Jupyter notebook (SI Dnonsense_calculations.pdf). Obtained results are presented in Excel spreadsheets (SI Dnonsense_calculations.xlsx) sheets showing results of the *dN/dS* parameter and *dN* with *dNONSENSE* comparison calculations for various studies. Frameshifts were excluded from the analysis, as mechanisms of such mutations differ from point mutations.

### Strains and media

Haploid *S. cerevisiae* strains used in this study were derivatives of W303. All primers used are listed in Table 1. To construct deletion strains *hog1Δ*, *pbs2Δ*, *rim15Δ*, *ure2Δ*, and *whi2Δ*, KanMX cassette conferring resistance to geneticin (G_418_) was amplified from the appropriate deletion haploid yeast strains from commercial collection (Euroscarf, Germany), along with ~ 200 bp of both ATG-upstream and stop codon-downstream DNA for homology. Wild-type strain W303 was transformed using the lithium acetate method and transformants were selected for G_418_ resistance^23^. All knockout strains were verified for correct cassette integration by PCR and sequencing of PCR-derived fragments. To eliminate potential non-relevant mutations that might appear due to applied genetic manipulations, the obtained deletion strains were backcrossed with the wild-type strain W303. All backcrossed diploid strains were subjected to sporulation, tetrad dissection, spore analysis, and replication onto a YPD medium containing geneticin (G_418_) to select for haploid progeny carrying respective gene deletion. Then again PCR verification of the desired genes’ presence and sequence verification were performed.

**Table 1:** Primers used in this study for the construction of deletion strains or correct cassette integration.

Primer name	Target PCR product	Sequence 5′ → 3′
731F_HOG1	*HOG1* or flanked KanMX*	TCGAAGGGAAGGAAGGAAAAA
2570R_HOG1		GACGGTTCTTGGAGTCTTAAAA
723F_PBS2	*PBS2* or flanked KanMX*	TGAGCCATACACGTTCTATAGA
3247R_PBS2		ATACTCTGTCATAATTCGTGCC
760F_RIM15	*RIM15* or flanked KanMX*	GTTGTTCGTATCACAGCATTTT
6558R_RIM15		AATTCTAATTTAGCCTCGAAAT
650F_URE2	*URE2* or flanked KanMX*	TAACAACATTAATCCGGGTGAC
2366R_URE2		TGCCGAGAAAAATACGCAAT
720F_WHI2	*WHI2* or flanked KanMX*	CCCCAAAGGTCATAAGAAGAT
2687R_WHI2		GGATGGGAAGATACGAAGAGA
AL1	Internal *KanMX* reverse primer	CGTGATTGCGCCTGAGCGAG

*KanMX cassette was flanked by upstream and downstream regions of the target gene.

The strains subjected to competitive experiments were grown in a complete synthetic liquid medium (0.67% yeast nitrogen base (YNB), 2% glycerol, 0.1% yeast extract, 0.1% glucose, required amino acids, and nucleotides) or YPD liquid medium (2% glucose, 1% yeast extract, 2% peptone, and enriched with 40 mg/L of adenine), depending on the medium used in an appropriate competition assay. Both media were additionally supplemented with ampicillin and streptomycin (25 μg/mL) to prevent bacterial contamination.

### Competition experiments

Competition tests were performed as follows: yeast were grown in an appropriate liquid medium overnight at 28 °C with shaking, adjusted to identical optical density, and then mixed at a 1:1 ratio in fresh medium (complete synthetic or YPD, respectively).

In terms of batch culture experiments, co-cultures were incubated with shaking at 28 °C for 24 h. A new round of subcultures was begun by transferring proper co-culture volumes into fresh medium to obtain OD600 = 0.1 and then growth was continued under the described above conditions. The procedures were repeated every 24 h. Aliquots of appropriate dilutions of each co-culture were plated in triplicate on YPD plates (every second day) and then the number of colonies of the wild-type and respective mutant strains were determined based on antibiotic resistance (deletion strains) or sensitivity (wild-type strain) by replicating them onto selective plates (YPD supplemented with 200 μg/ml of geneticin (G_418_)).

In terms of continuous culture experiments, co-cultures were incubated with shaking at 28 °C for 24 h and then placed in a self-made continuous culture set described previously^19^. The flow of the medium was turned on at a dilution rate of 0.17–0.18 vol/h (complete synthetic medium) or 0.36–0.38 vol/h (YPD medium). Each strain was cultivated in triplicate. In total, 15 chambers were inoculated. Culture samples were passively collected and every second day, OD_600_ measurements were taken, aliquots of appropriate dilutions of each co-culture were plated in triplicate on YPD plates, and grown colonies were tested for G_418_ resistance.

### Phylogenetics

We determined the phylogenetic distribution of functional orthologs of mutated genes using KEGG Orthology (KO) tables.

### Supplementary Information

Supplementary Information 1.

Supplementary Information 2.

## Data Availability

All data generated or analysed during this study are included in this article and supplementary information files.
