# Supplementary material for: Loss-of-function mutations are main drivers of adaptations during short-term evolution
Source: Sci Rep. 2024 Mar 26;14:7128. doi: 10.1038/s41598-024-57694-8 (PMC10965932; doi:10.1038/s41598-024-57694-8)
Supplement: Supplementary file 1 — Supplementary Information 1. [file 41598_2024_57694_MOESM1_ESM.pdf]

In [1]:

```
#These commands import python libraries.
from Bio import SeqIO
from Bio.Seq import Seq
from Bio.SeqRecord import SeqRecord
import sys
import os
import numpy as np
from scipy.stats import chi2_contingency
import pandas as pd
import re
```

In [2]:

```
#These command imports pygenome library applied for importing yeast sequences.
import pygenome
from pygenome import saccharomyces_cerevisiae as sg
```

In [3]:

```
#The function add_fasta_sequence_long_name imports sequences using long ID

def add_fasta_sequence_long_name(input_name):
    mygene=sg.sysgenes[input_name]
    mygene1=str(mygene.cds().seq)[:-3]
    return(mygene1)
```

In [4]:

```
from collections import Counter
```

In [5]:

```
#This function calculates number of synonymous, non-synonymous and nonsense position
#protein coding sequence

def gene_nonsense_ds(input_dna):
    synonymous_number=0
    nonsense_number=0
    non_synonymous_number=0
    dna_code=str("ACTG")
    input_bialko=Seq(input_dna).translate()
    for i in range(0,len(input_dna)):
    #mutated_str=input_dna
        #print(dna_code)
        for i1 in dna_code:
            mutated_str=list(input_dna)
            if input_dna[i]!=i1:
                mutated_str[i]=i1
                mutated_dna="".join(mutated_str)
                mutated_bialko=Seq(mutated_dna).translate()
            #print(mutated_dna,mutated_bialko)
                if mutated_bialko==input_bialko:
                #print("synonymous")
                #print(mutated_dna,mutated_bialko)
                   synonymous_number=synonymous_number+1
            #print(wynik1)
                if mutated_bialko.count("*")>0:
                #print(mutated_bialko.count("*"))
                #print(mutated_dna,mutated_bialko)
                    nonsense_number=nonsense_number+1
                if  mutated_bialko!=input_bialko:
                    if mutated_bialko.count("*")==0:
                         #print("mutacja")
                         #print(mutated_dna,mutated_bialko)
                         non_synonymous_number=non_synonymous_number+1
```

```
    return(synonymous_number,nonsense_number,non_synonymous_number)
lista_genow_sg=list(sg.sysgenes.keys())

#This function calculates number of synonymous, non-synonymous and nonsense position
#in sequences encoded by list of gene names.

def gene_nonsense_ds_lista_long(lista_genow1):
       liczba_genow=0
#lista_genow=["XKS1","XKS1"]
       lista_genow=list(set(lista_genow1))
       wypluj=""
       tb=[0,0,0]
       tablica_wynikow=np.array(tb)
       for gene in lista_genow:
            print(gene)
            #wypluj=wypluj+add_fasta_sequence(gene)
            if(lista_genow_sg.count(gene)>0):
                tp=np.array(gene_nonsense_ds(add_fasta_sequence_long_name(gene)))
                nn=lista_genow1.count(gene)
                print(tp*nn)
                tablica_wynikow=tablica_wynikow+tp*nn
                liczba_genow=liczba_genow+1
            else:
                print("wykluczony gen",gene)
            #print(nn)
            #tablica_wynikow0=tablica_wynikow0+(tablica_wynikow[0]*2)
            #tablica_wynikow1=tablica_wynikow1+(tablica_wynikow[1]*2)
            #tablica_wynikow2=tablica_wynikow2+(tablica_wynikow[2]*2)
       suma=tablica_wynikow[0]+tablica_wynikow[1]+tablica_wynikow[2]
       print("synonymous sides ",tablica_wynikow[0])
       print("nonsense sides ",tablica_wynikow[1])
       print("missense sides ",tablica_wynikow[2])
       print("liczba genow",liczba_genow)
#sg.sysgenes["YHR054C-B"]
lista_genow=["YGR194C","YHR054C-B"]
gene_nonsense_ds_lista_long(lista_genow)
#lista_genow_sg=list(sg.sysgenes.keys())
#lista_genow_sg.count("YHR054C-B")
```

```
YHR054C-B
wykluczony gen YHR054C-B
YGR194C
[1144  261 3995]
synonymous sides  1144
nonsense sides  261
missense sides  3995
liczba genow 1
```

In [6]:

```
#These commends imports all yeast gene names.

#YPR099C YBR266C were excluded, as it doesn't work
df=pd.read_csv('Lista_wszystkich_genów.txt',sep='\t')
df.drop('5', axis=1, inplace=True)
df.drop('6', axis=1, inplace=True)
df.drop('7', axis=1, inplace=True)
df.drop('1', axis=1, inplace=True)
df.drop('2', axis=1, inplace=True)
df.drop('8', axis=1, inplace=True)
df.drop('4', axis=1, inplace=True)
df.drop('10', axis=1, inplace=True)
df.drop('11', axis=1, inplace=True)
df.drop('12', axis=1, inplace=True)
#df.drop('9', axis=1, inplace=True
df1=df.dropna(how='all')
```

```python
#os.getcwd()
#os.chdir('Wiele_analiz_do_pracy_o_ewolucji')
df2=df1[df1['3'].str.contains("^Y")]
lista_wszystkich_genow=df2['3'].values.tolist()
gene_nonsense_ds_lista_long(lista_wszystkich_genow)
```

```
YBR229C
[1768  398 6420]
YMR243C
[ 903  167 2908]
YIL120W
[1167  242 3658]
YIL129C
[ 4562  1075 15747]
YIR032C
[ 380   78 1297]
YOR126C
[ 443  106 1593]
YDR253C
[ 349   82 1288]
YHR070W
[ 965  181 3345]
YCR068W
[1042  234 3404]
YDL043C
[ 484  127 1783]
YBL013W
[ 852  128 2629]
YIL172C
[1051  279 3971]
YDL171C
[ 4229   916 14160]
YLR116W
[ 960  200 3124]
YCR046C
[ 355   74 1092]
YJL187C
[1549  339 5483]
YPL223C
[ 301   68 1143]
YHR047C
[1624  379 5701]
YAL017W
[2589  564 9051]
YJR070C
[ 638  131 2156]
YAR003W
[ 804  190 2840]
YGR179C
[ 729  201 2724]
YMR097C
[ 707  167 2429]
YKR041W
[ 447  101 1702]
YPL167C
[2808  741 9987]
YMR247C
[ 2891   742 10425]
YBR060C
[1161  272 4147]
YOR064C
[ 393  109 1469]
YBR165W
[ 488  120 1885]
```

```
YIL114C
[ 576  107 1846]
YJL061W
[1283  318 4816]
YML125C
[ 624  137 2047]
YML114C
[ 973  200 3417]
YDR372C
[ 639  171 2295]
YPR005C
[ 553  138 1955]
YLR288C
[ 923  201 3142]
YGR103W
[1064  303 4078]
YNL201C
[1567  388 5767]
YDR064W
[305  68 986]
YOR293W
[180  71 694]
YBR133C
[1590  380 5473]
YJR093C
[ 627  103 2213]
YCR014C
[1018  314 3906]
YFR009W
[1421  353 4994]
YDR200C
[1167  271 3998]
YGL163C
[1712  400 5970]
YBR139W
[ 956  229 3387]
YOR304C-A
[138  31 515]
YOR020W-A
[170  35 605]
YPL031C
[ 605  128 2012]
YOL021C
[1951  414 6644]
YIL088C
[1012  209 3189]
YDL072C
[ 407   78 1342]
YFR043C
[ 415  103 1615]
YOR353C
[1548  329 5242]
YGR276C
[1037  235 3705]
YGL170C
[ 715  203 2799]
YPL143W
[218  45 700]
YOR208W
[1380  357 5013]
YGR261C
[1550  393 5338]
YNL117W
[1064  230 3692]
```

```
YPR124W
[ 722  158 2774]
YKR013W
[ 749   96 2116]
YPR017C
[252  55 980]
YDR383C
[ 435  119 1588]
YJR022W
[200  43 738]
YMR261C
[2008  446 7032]
YDR316W
[ 826  255 3158]
YML012W
[ 415   92 1392]
YNR067C
[2315  518 7220]
YGL253W
[ 943  187 3244]
YBL078C
[222  49 782]
YLR390W-A
[ 552   71 1519]
YDR086C
[152  35 533]
YKL065C
[ 398   99 1357]
YHL021C
[ 896  191 3098]
YOR313C
[ 645  153 2244]
YBR282W
[283  79 952]
YML051W
[ 871  190 2854]
YNL094W
[1145  229 3909]
YER022W
[1238  296 4649]
YKL154W
[ 489  110 1597]
YCL032W
[ 654  152 2308]
YJL034W
[1351  264 4523]
YKL141W
[ 420   95 1267]
YKL210W
[1996  389 6831]
YGL200C
[ 392   77 1358]
YMR168C
[1111  296 4065]
YIL093C
[ 503  127 1746]
YOR357C
[ 297   69 1092]
YOL152W
[1204  281 4095]
YOR220W
[ 532  123 1730]
YLR437C
[261  60 876]
```

YKL191W
[1073 211 3522]
YGR123C
[943 211 3463]
YNL309W
[857 148 2775]
YMR043W
[504 143 1927]
YMR133W
[812 174 2866]
YJL102W
[1617 343 5411]
YJR123W
[455   83 1487]
YHL032C
[1421 293 4667]
YNL204C
[536 120 2044]
YGR147C
[547 117 1928]
YKL029C
[1387 265 4369]
YPL266W
[619 137 2106]
YMR186W
[1287 339 4719]
YOL003C
[700 193 2509]
YBR162W-A
[132 29 424]
YMR307W
[1122 200 3709]
YBR137W
[325   91 1195]
YPL047W
[199 42 650]
YGR192C
[687 109 2192]
YDL121C
[282 65 994]
YOR230W
[860 173 2900]
YPL059W
[300 68 982]
YJR135C
[446 108 1597]
YNL161W
[1377 392 5035]
YPL137C
[2547 511 8426]
YMR263W
[351   69 1389]
YGL158W
[954 237 3417]
YNL243W
[1890 421 6401]
YNL328C
[295 60 959]
YHR052C-B
wykluczony gen YHR052C-B
YNL066W
[927 156 2697]
YOL064C
[712 165 2336]

YLR028C
[1190 256 3873]
YGL026C
[1465 259 4639]
YKL206C
[490 131 1782]
YOL077C
[533 142 1944]
YNL284C
[638 149 2111]
YDR088C
[666 209 2563]
YLR250W
[413   94 1599]
YJR042W
[1392 367 4937]
YGL133W
[2294 600 8482]
YIL085C
[893 250 3510]
YDR502C
[757 167 2532]
YGL033W
[390 104 1432]
YGR132C
[582 123 1878]
YER031C
[425   96 1486]
YBR042C
[766 188 2619]
YLR107W
[757 176 2703]
YLR079W
[557 108 1891]
YLR284C
[520 131 1869]
YOR231W
[996 245 3331]
YGL130W
[848 225 3058]
YJL093C
[1370 295 4554]
YDR462W
[282 73 968]
YBR055C
[1653 485 5953]
YOR182C
[132 28 407]
YKL197C
[1986 457 6944]
YAL008W
[385   85 1312]
YLR347C
[1698 351 5700]
YDR297W
[637 141 2363]
YIR009W
[217 39 743]
YGR163W
[647 141 2281]
YNR031C
[2946   700 10565]
YLR386W
[1673 411 5836]

YDR233C
[575 128 1952]
YKL084W
[197 57 790]
YKL009W
[454 124 1546]
YAR007C
[1164 242 4183]
YGL207W
[1906 482 6927]
YOR180C
[529 106 1804]
YBL032W
[772 124 2533]
YKL023C-A
[152 31 492]
YLR438C-A
[180 33 588]
YNL317W
[863 201 3121]
YPR193C
[271   68 1065]
YDR306C
[906 254 3142]
YNL073W
[1097 260 3827]
YER034W
[351   76 1238]
YDR125C
[812 235 3030]
YOR264W
[838 179 2853]
YLR412W
[507 136 1823]
YPL119C
[1237 248 4068]
YNL225C
[1048 293 3888]
YNR052C
[795 195 2907]
YML127W
[1173 247 3809]
YPL094C
[517 132 1817]
YNL196C
[535 141 2006]
YMR294W
[642 192 2523]
YDR025W
[315   62 1027]
YPL225W
[252 76 986]
YDR497C
[1218 221 3817]
YLR375W
[673 134 2280]
YOR388C
[741 160 2483]
YBR272C
[898 229 3193]
YMR315W
[698 132 2311]
YPL117C
[498 131 1963]

YOR214C
[471 104 1549]
YPL055C
[626 151 2211]
YPL170W
[296   46 1026]
YKR083C
[227 72 898]
YPL203W
[733 174 2513]
YBR268W
[197 55 693]
YOR133W
[1658 325 5595]
YML063W
[489 109 1697]
YBR009C
[224 46 657]
YHR107C
[776 178 2709]
YDR059C
[298 70 964]
YMR088C
[1152 262 3644]
YDR227W
[2589 592 9041]
YGL137W
[1735 405 5861]
YML103C
[3142   798 10955]
YJL190C
[251 53 866]
YER057C
[263 42 856]
YBR035C
[436 100 1516]
YDL012C
[193 73 697]
YNL076W
[1117 236 3903]
YMR162C
[3150   721 11033]
YIL098C
[280   82 1033]
YKL081W
[788 181 2739]
YMR309C
[1496 402 5410]
YLL041C
[510 136 1748]
YGR197C
[1087 240 3596]
YMR009W
[329   90 1192]
YGR076C
[281   90 1042]
YBL002W
[275 55 849]
YGR031W
[352   52 1063]
YPL037C
[297   60 1056]
YGR199W
[1425 382 5024]

```
YKL198C
[1386  216 4356]
YJL028W
[244  39 716]
YGL233W
[1634  429 6127]
YGL164C
[ 809  203 2948]
YPL233W
[ 371  112 1461]
YNL254C
[ 748  204 2657]
YLR291C
[ 763  145 2521]
YPL125W
[1924  543 6821]
YCR032W
[ 3955  1024 14524]
YOR091W
[ 619  154 2332]
YDR198C
[ 873  271 3167]
YHR169W
[ 874  172 2833]
YBR129C
[ 575  137 2240]
YIL009W
[1350  291 4605]
YER006W
[1025  225 3430]
YHR121W
[ 346   86 1251]
YNL290W
[ 672  135 2253]
YNL081C
[275  53 959]
YNR057C
[ 478   91 1564]
YGR125W
[2036  474 6814]
YPL011C
[ 661  161 2355]
YKL167C
[244  57 932]
YDL115C
[ 614  156 2407]
YJR121W
[1096  200 3303]
YDR321W
[ 760  151 2518]
YOR197W
[ 817  196 2875]
YGL003C
[1163  232 3699]
YDR267C
[ 632  154 2184]
YOR090C
[1156  250 3742]
YDL164C
[1454  349 4992]
YOR360C
[ 976  233 3525]
YDR495C
[1804  486 6809]
```

```
YPR129W
[ 658  148 2335]
YHR062C
[ 593  104 1940]
YBR140C
[ 5942  1422 20464]
YML100W
[2165  438 7279]
YBR180W
[1168  231 3749]
YLR394W
[ 960  218 3160]
YML092C
[ 533  112 1605]
YPL109C
[1247  295 4371]
YHR157W
[ 334   77 1227]
YMR115W
[ 905  267 3337]
YPL018W
[ 694  162 2465]
YBR085C-A
[162  41 562]
YER087W
[1043  289 3852]
YBR028C
[ 998  243 3484]
YLR234W
[1306  270 4328]
YOR189W
[210  57 777]
YIL116W
[ 765  175 2525]
YLR223C
[1892  462 7411]
YNL282W
[ 380   84 1291]
YPR083W
[1100  259 3852]
YAL049C
[ 470   97 1647]
YPL049C
[ 890  178 3000]
YDR188W
[1109  195 3610]
YPR015C
[ 500  110 1613]
YPR023C
[ 757  197 2655]
YOL133W
[196  56 837]
YBR054W
[ 673  139 2284]
YDR017C
[1931  480 7039]
YGL213C
[ 767  183 2623]
YHR124W
[1200  306 4137]
YMR070W
[ 879  160 3371]
YDR510W
[181  49 679]
```

```
YJL178C
[ 514  111 1814]
YGL143C
[ 816  187 2714]
YOR192C
[1158  239 3994]
YFL036W
[2590  645 8924]
YBR160W
[ 573  141 1968]
YNL224C
[1401  320 5182]
YIL126W
[2510  668 9053]
YEL007W
[1303  229 4462]
YEL040W
[ 948  194 3061]
YPL235W
[ 949  190 3100]
YPL082C
[ 3684   823 12296]
YBR106W
[ 344   68 1280]
YKL048C
[1223  293 4244]
YHR190W
[ 807  218 2971]
YDR280W
[ 597  114 2034]
YNL223W
[ 931  205 3310]
YDR351W
[1580  415 5781]
YDL193W
[ 709  180 2486]
YLR094C
[ 991  196 3331]
YGL083W
[1538  382 5316]
YOR356W
[1239  313 4127]
YER137C
[268  75 989]
YGL086W
[1253  403 5085]
YGR236C
[184  43 628]
YDR098C
[ 454  118 1678]
YGR288W
[ 903  219 3135]
YCR093W
[ 4055   964 13953]
YLR425W
[2470  582 8711]
YNL022C
[ 910  216 3284]
YGL154C
[ 512  112 1824]
YKR023W
[ 929  281 3560]
YNL145W
[ 72  14 256]
```

YGL176C
[1063 244 3679]
YPR199C
[557 113 1976]
YDR472W
[570 122 1855]
YPL169C
[1152 252 3987]
YLR015W
[910 264 3371]
YJL098W
[1876 472 7174]
YLL022C
[718 150 2597]
YLR411W
[444 130 1595]
YDR335W
[2230 597 8189]
YDL154W
[1691 407 6011]
YHL009C
[579 149 2242]
YML021C
[689 169 2373]
YAL056W
[1672 371 5877]
YOR216C
[842 246 3268]
YPR018W
[1077 310 4067]
YOL113W
[1227 313 4355]
YDL046W
[360   64 1133]
YKR104W
[611 141 2002]
YHR066W
[850 206 3021]
YAL058W
[979 210 3329]
YCL018W
[754 131 2391]
YIR021W
[729 139 2399]
YER007W
[948 227 3487]
YAL031C
[1507 361 4972]
YGR056W
[1745 403 6204]
YNL003C
[592 120 1844]
YPR079W
[744 163 2522]
YLR321C
[766 160 2908]
YBR166C
[854 215 2999]
YNL295W
[965 251 3500]
YHR005C-A
[167 45 625]
YDR397C
[272 63 979]

```
YDR416W
[1572  468 5691]
YNR060W
[1430  330 4711]
YHL010C
[1032  284 3949]
YBR299W
[1042  268 3946]
YLL015W
[ 3067   689 10275]
YOR361C
[1413  318 5136]
YJR035W
[2013  529 7223]
YDR035W
[ 708  152 2470]
YOL030W
[ 994  187 3175]
YOR378W
[1065  194 3376]
YLL031C
[1986  436 6731]
YGR133W
[ 345   71 1231]
YKL013C
[ 325   73 1141]
YOL052C-A
[142  11 396]
YIR028W
[1244  266 4205]
YFL003C
[1665  384 5853]
YOR311C
[ 568  118 1924]
YKL043W
[ 755  137 2402]
YLR427W
[1248  297 4485]
YMR213W
[1108  274 3928]
YPR060C
[ 475  123 1706]
YOR283W
[ 428  105 1537]
YHR086W
[1020  214 3473]
YBR078W
[ 923  111 2827]
YER164W
[2712  698 9802]
YPL209C
[ 684  172 2447]
YPR098C
[ 329   74 1046]
YLR078C
[ 471  115 1610]
YAL048C
[1280  289 4389]
YAL025C
[ 509  168 2077]
YOR288C
[ 594  148 2120]
YPL181W
[ 955  227 3372]
```

```
YHL019C
[1086  257 4102]
YMR073C
[ 376   95 1338]
YJR108W
[231  52 824]
YCR002C
[ 623  129 2146]
YLR070C
[ 743  139 2322]
YCR010C
[ 584  112 1851]
YLR056W
[ 687  157 2441]
YIR014W
[ 482  104 1592]
YOR270C
[1572  374 5614]
YPL133C
[ 866  195 2953]
YPL184C
[1153  237 4118]
YLR262C-A
[110  30 436]
YAL014C
[ 512  106 1677]
YMR060C
[ 607  179 2157]
YCL030C
[1597  320 5274]
YOR086C
[2274  549 7851]
YDR244W
[1104  284 4120]
YDL089W
[ 870  237 3249]
YHR080C
[2573  555 8977]
YER099C
[ 647  122 2093]
YJL045W
[1307  258 4141]
YMR211W
[ 855  230 3190]
YLR068W
[ 257   90 1012]
YMR173W
[ 749  168 2953]
YDR322C-A
[184  45 635]
YHR010W
[266  60 898]
YGR240C
[2000  383 6500]
YNL132W
[2041  478 6985]
YNL123W
[2006  390 6577]
YHL014C
[ 756  174 2715]
YLR352W
[1515  381 5367]
YPL124W
[ 483  123 1671]
```

YER069W
[1766 355 5646]
YKR066C
[704 151 2394]
YPL221W
[1554 333 5250]
YBL084C
[1441 343 5038]
YCL058W-A
[211 53 753]
YMR114C
[654 195 2463]
YDR490C
[1507 317 5070]
YIL053W
[472 102 1676]
YPL279C
[750 148 2477]
YAR071W
[864 200 3139]
YJL012C
[1388 314 4787]
YML101C
[247 32 774]
YJL089W
[1477 400 5584]
YPR056W
[675 157 2210]
YDL174C
[1140 231 3912]
YBL030C
[655 138 2069]
YER112W
[302   71 1310]
YLR403W
[1294 212 4641]
YOR348C
[1321 224 4098]
YDL153C
[1054 307 4129]
YLR172C
[578 142 1980]
YNL100W
[452 127 1527]
YNR073C
[994 198 3326]
YOL052C
[716 181 2667]
YLR209C
[633 116 2050]
YLL057C
[805 182 2721]
YPL148C
[330   89 1138]
YGL183C
[370 124 1477]
YCR066W
[900 226 3257]
YML128C
[893 262 3462]
YOL145C
[1919 560 7214]
YDL175C
[629 165 2302]

```
YGR183C
[128  29 437]
YGR245C
[1421  354 5128]
YDR331W
[ 769  157 2773]
YHR132W-A
[252  59 868]
YOR046C
[ 939  206 3193]
YDL169C
[ 390   99 1518]
YOL140W
[ 826  178 2803]
YIL089W
[ 395   91 1359]
YIL068C
[1469  383 5393]
YGR178C
[1365  274 4859]
YGR193C
[ 805  182 2703]
YCR094W
[ 720  170 2629]
YER156C
[ 642  133 2267]
YDL235C
[ 301   72 1130]
YGR037C
[156  50 577]
YKR079C
[1513  356 5673]
YNL294C
[1023  237 3537]
YAL063C
[3071  353 8474]
YOL034W
[1986  526 7325]
YMR044W
[ 835  225 3215]
YNL107W
[ 408   96 1530]
YHR017W
[ 747  190 2528]
YDR314C
[1227  365 4636]
YDR201W
[ 300   82 1103]
YHR087W
[223  44 732]
YOR010C
[ 589   73 1597]
YFL047W
[1319  327 4780]
YHR108W
[1145  251 3869]
YLR395C
[144  34 524]
YMR142C
[ 401   86 1304]
YLR295C
[246  57 813]
YLR414C
[ 534  113 1720]
```

```
YKR011C
[ 618  187 2372]
YOR021C
[ 388   99 1430]
YLR393W
[ 518  122 1871]
YPL091W
[ 959  192 3196]
YJL050W
[2030  486 7141]
YPL273W
[ 607  134 2184]
YOR212W
[ 802  197 2808]
YDR084C
[ 390   93 1308]
YIL017C
[1712  429 6148]
YDR218C
[ 771  195 2841]
YDL180W
[1040  227 3656]
YEL071W
[ 953  207 3304]
YLR186W
[ 510  103 1655]
YLR406C
[224  53 740]
YDL093W
[1437  342 4908]
YDR423C
[ 780  181 2720]
YGR134W
[2069  512 7517]
YGL112C
[1044  221 3379]
YDR236C
[ 436   86 1440]
YKR034W
[ 540  118 1763]
YEL037C
[ 839  154 2589]
YJL048C
[ 755  187 2622]
YJL116C
[ 698  130 2205]
YOL156W
[1178  217 3708]
YGL113W
[1274  339 4399]
YPR085C
[ 832  198 2957]
YDL013W
[1218  250 4103]
YML007W
[1161  256 4433]
YDR138W
[1337  365 5066]
YOR141C
[1622  386 5921]
YPL131W
[ 583  144 1946]
YDR288W
[ 562  137 2028]
```

```
YDL001W
[ 787  206 2877]
YMR318C
[ 701  159 2380]
YGR106C
[ 486  117 1782]
YLR262C
[ 390  117 1428]
YBR087W
[ 694  166 2326]
YBR302C
[ 678  192 2541]
YOR173W
[ 643  162 2372]
YGR159C
[ 837  174 2715]
YBR015C
[1138  267 3968]
YKL023W
[ 487  139 1867]
YML048W
[ 768  172 2687]
YHR016C
[ 968  180 3064]
YHR216W
[1066  198 3443]
YMR266W
[1855  421 6301]
YHR019C
[1081  250 3655]
YGR017W
[ 516  131 2026]
YEL046C
[ 779  148 2556]
YMR170C
[1025  200 3329]
YGL196W
[ 831  191 2830]
YKL152C
[ 477  122 1624]
YLR043C
[198  35 694]
YNL264C
[ 648  156 2346]
YPR192W
[ 652   99 1994]
YDR252W
[ 271   62 1008]
YGR104C
[ 586  131 2046]
YGR155W
[1014  203 3346]
YLL046C
[ 445  104 1692]
YDR044W
[ 626  145 2181]
YIL138C
[ 246  108 1095]
YJR086W
[199  64 727]
YLR113W
[ 849  160 2906]
YNL221C
[1661  418 5796]
```

```
YCL004W
[1034  259 3396]
YBR233W
[ 829  145 2743]
YBL079W
[2893  651 9974]
YDR054C
[ 507  112 2036]
YJL153C
[1035  215 3547]
YDR308C
[253  65 942]
YGL212W
[ 611  141 2092]
YGR112W
[ 760  187 2554]
YDR456W
[1283  242 4172]
YDR483W
[ 788  221 2969]
YLR346C
[186  52 671]
YDL107W
[ 604  180 2375]
YOR007C
[ 656  149 2309]
YBR041W
[1267  319 4435]
YKL204W
[1186  268 4234]
YDL022W
[ 778  155 2586]
YPL022W
[2057  527 7316]
YDR493W
[246  54 807]
YGL011C
[ 501  106 1661]
YBR260C
[1267  283 4444]
YNL065W
[1170  250 3854]
YLR190W
---------------------------------------------------------------------------
KeyboardInterrupt                         Traceback (most recent call last)
~\AppData\Local\Temp/ipykernel_61188/4209973768.py in <module>
     19 df2=df1[df1['3'].str.contains("^Y")]
     20 lista_wszystkich_genow=df2['3'].values.tolist()
---> 21 gene_nonsense_ds_lista_long(lista_wszystkich_genow)

~\AppData\Local\Temp/ipykernel_61188/718795429.py in gene_nonsense_ds_lista_long(list
a_genow1)
     49             #wypluj=wypluj+add_fasta_sequence(gene)
     50             if(lista_genow_sg.count(gene)>0):
---> 51                 tp=np.array(gene_nonsense_ds(add_fasta_sequence_long_name(gen
e)))
     52                 nn=lista_genow1.count(gene)
     53                 print(tp*nn)

~\AppData\Local\Temp/ipykernel_61188/718795429.py in gene_nonsense_ds(input_dna)
     16                 mutated_str[i]=i1
     17                 mutated_dna="".join(mutated_str)
---> 18                 mutated_bialko=Seq(mutated_dna).translate()
     19             #print(mutated_dna,mutated_bialko)
```

```
     20                 if mutated_bialko==input_bialko:

~\anaconda3\lib\site-packages\Bio\Seq.py in translate(self, table, stop_symbol, to_st
op, cds, gap)
   1375
   1376         return self.__class__(
-> 1377             _translate_str(str(self), table, stop_symbol, to_stop, cds, gap=g
ap)
   1378         )
   1379

~\anaconda3\lib\site-packages\Bio\Seq.py in _translate_str(sequence, table, stop_symb
ol, to_stop, cds, pos_stop, gap)
   2992         codon = sequence[i : i + 3]
   2993         try:
-> 2994             amino_acids.append(forward_table[codon])
   2995         except (KeyError, CodonTable.TranslationError):
   2996             if codon in codon_table.stop_codons:

~\anaconda3\lib\site-packages\Bio\Data\CodonTable.py in __getitem__(self, codon)
    426         """
    427         try:
--> 428             x = self._cache[codon]
    429         except KeyError:
    430             pass

KeyboardInterrupt:
```

synonymous sides 5058099

nonsense sides 1166436

missense sides 17554023

In [ ]:

```
#https://www.nature.com/articles/s41586-022-04823-w#MOESM5
#Synonymous mutations in representative yeast genes are mostly strongly non-neutral
#Synonymous_non-neutral_MOESM5_ESM.xls
#Ten plik pokazuje, że prawie wszystkie nonsense są deleterious
```

In [7]:

```
#This is a function for calculating probability that nonsense and missense substitio

def statystyka_nonsense_essential_included(synony,nonsen,missense):
#lista_genow=["XKS1","XKS1"]
        tablica_wynikow=[5058099,1166436,17554023]
        ds_rate=synony/tablica_wynikow[0]
        d_nonsense_rate=nonsen/tablica_wynikow[1]
        dn_rate=missense/tablica_wynikow[2]
        if(ds_rate>0):
             dn_to_ds=dn_rate/ds_rate
             dnonsense_to_ds=d_nonsense_rate/ds_rate
        else:
             dn_to_ds="error ds=0"
             dnonsense_to_ds="error ds=0"

        print("synonymous sides ",tablica_wynikow[0], " synonymous mutations ",synony
        print("nonsense sides ",tablica_wynikow[1], " nonsense mutations ",nonsen,"dn
        tab_data = [[synony,tablica_wynikow[0]], [nonsen,tablica_wynikow[1]]]
```

```
        pvalue=chi2_contingency(tab_data)[1]
        print("P-value nonsense mutation rate=synonymous mutation rate",pvalue)
        print("missense sides ",tablica_wynikow[2], " missense mutations ",missense,"
        tab_data = [[synony,tablica_wynikow[0]], [missense,tablica_wynikow[2]]]
        pvalue=chi2_contingency(tab_data)[1]
        print("P-value missense mutation rate=synonymous mutation rate",pvalue)
        print("dNONSENSE/dS ",dnonsense_to_ds)
        print("dN/dS ",dn_to_ds)
#This is a function for calculating probability that nonsense and missense substitio
#assuming that the puryfying selection removes 80% of nonsense mutations.

def statystyka_nonsense_deleterious_excluded(synony,nonsen,missense):
#lista_genow=["XKS1","XKS1"]
        tablica_wynikow=[5058099,233292,17554023]
        ds_rate=synony/tablica_wynikow[0]
        d_nonsense_rate=nonsen/tablica_wynikow[1]
        dn_rate=missense/tablica_wynikow[2]
        if(ds_rate>0):
             dn_to_ds=dn_rate/ds_rate
             dnonsense_to_ds=d_nonsense_rate/ds_rate
        else:
             dn_to_ds="error ds=0"
             dnonsense_to_ds="error ds=0"
        print("The corrected dNONSENSE/dS calculations")
        print("synonymous sides ",tablica_wynikow[0], " synonymous mutations ",synony
        print("nonsense sides ",tablica_wynikow[1], " nonsense mutations ",nonsen,"dn
        tab_data = [[synony,tablica_wynikow[0]], [nonsen,tablica_wynikow[1]]]
        pvalue=chi2_contingency(tab_data)[1]
        print("P-value nonsense mutation rate=synonymous mutation rate",pvalue)
        #print("missense sides ",tablica_wynikow[2], " missense mutations ",missense,
        #tab_data = [[synony,tablica_wynikow[0]], [missense,tablica_wynikow[2]]]
        #pvalue=chi2_contingency(tab_data)[1]
        #print("P-value missense mutation rate=synonymous mutation rate",pvalue)
        print("dNONSENSE/dS ",dnonsense_to_ds)
        #print("dN/dS ",dn_to_ds)
```

In [8]:

```
#The calculation of statistics for the study "Adaptive evolution of engineered yeast
#https://onlinelibrary.wiley.com/doi/10.1002/yea.3559  (see table 2)
# perhaps there is a bias, as mutations are selected by authors
#synonymous 22 nonsense 3 missense 47
#Only metabolism related genes
#statystyka_nonsense_essential_excluded(22,3,47)
#statystyka_nonsense_deleterious_excluded(22,3,47)
statystyka_nonsense_essential_included(22,3,47)
statystyka_nonsense_deleterious_excluded(22,3,47)
```

```
synonymous sides  5058099  synonymous mutations  22 ds 4.34946014303002e-06
nonsense sides  1166436  nonsense mutations  3 dnonsense 2.5719370801312716e-06
P-value nonsense mutation rate=synonymous mutation rate 0.5436844310376838
missense sides  17554023  missense mutations  47 dn 2.677448924386165e-06
P-value missense mutation rate=synonymous mutation rate 0.0797322818462641
dNONSENSE/dS  0.5913232896852229
dN/dS  0.6155818966813061
The corrected dNONSENSE/dS calculations
synonymous sides  5058099  synonymous mutations  22 ds 4.34946014303002e-06
nonsense sides  233292  nonsense mutations  3 dnonsense 1.2859420811686642e-05
P-value nonsense mutation rate=synonymous mutation rate 0.17328381796778708
dNONSENSE/dS  2.9565556158259723
```

In [9]:

```
#The calculation of statistics for the study "Aneuploidy Underlies Rapid Adaptive Ev
#https://www.sciencedirect.com/science/article/pii/S0092867408011963
#The Excel sheet was imported from SOM. The number of substitutions was calculated u
```

```
#Although different clones were sequencing, most mutations aquired during experiment
df=pd.read_excel('1-s2.0-S0092867408011963-mmc10.xls',skiprows=3)
nonsense=df['Impact'].str.contains('nonsense').value_counts()[True]
synonymous=df['Impact'].str.contains('^synonymous').value_counts()[True]
missense=df['Impact'].str.contains('[A-Z][0-9]+[A-Z]').value_counts()[True]

statystyka_nonsense_essential_included(synonymous,nonsense,missense)
statystyka_nonsense_deleterious_excluded(synonymous,nonsense,missense)
nonsense,missense,synonymous
```

```
synonymous sides  5058099  synonymous mutations  322 ds 6.366028027525756e-05
nonsense sides  1166436  nonsense mutations  21 dnonsense 1.8003559560918902e-05
P-value nonsense mutation rate=synonymous mutation rate 3.244304517350907e-09
missense sides  17554023  missense mutations  391 dn 2.2274096370957246e-05
P-value missense mutation rate=synonymous mutation rate 5.0927449651679436e-48
dNONSENSE/dS  0.28280679071901965
dN/dS  0.3498900142231133
The corrected dNONSENSE/dS calculations
synonymous sides  5058099  synonymous mutations  322 ds 6.366028027525756e-05
nonsense sides  233292  nonsense mutations  21 dnonsense 9.001594568180649e-05
P-value nonsense mutation rate=synonymous mutation rate 0.15727888737771098
dNONSENSE/dS  1.4140048597428563
```

Out[9]:
```
(21, 391, 322)
```

In [10]:
```
#The calculation of statistics for the study "Microfluidic screening and whole-genom
#https://www.pnas.org/doi/full/10.1073/pnas.1506460112
#Authors of this study wrote: 'Among the 330 point mutations, 248 mutations were in
statystyka_nonsense_essential_included(79,18,151)
statystyka_nonsense_deleterious_excluded(79,18,151)
```

```
synonymous sides  5058099  synonymous mutations  79 ds 1.5618515968153252e-05
nonsense sides  1166436  nonsense mutations  18 dnonsense 1.543162248078763e-05
P-value nonsense mutation rate=synonymous mutation rate 1.0
missense sides  17554023  missense mutations  151 dn 8.602016757070444e-06
P-value missense mutation rate=synonymous mutation rate 1.8658138463336022e-05
dNONSENSE/dS  0.9880338511196132
dN/dS  0.5507576247711552
The corrected dNONSENSE/dS calculations
synonymous sides  5058099  synonymous mutations  79 ds 1.5618515968153252e-05
nonsense sides  233292  nonsense mutations  18 dnonsense 7.715652487011985e-05
P-value nonsense mutation rate=synonymous mutation rate 6.159121283802781e-11
dNONSENSE/dS  4.940067611253523
```

In [11]:
```
#The calculation of statistics for the study: "Pervasive genetic hitchhiking and clo
#https://www.nature.com/articles/nature12344#MOESM19
#The authors presented mutations for all experiments together.
#The Excel sheet was imported from SOM. The number of substitutions was calculated u
#
df=pd.read_excel('41586_2013_BFnature12344_MOESM20_ESM.xlsx',skiprows=3)
synonymous=df['Class.1'].str.contains('^Syn').value_counts()[True]
nonsense=(df['Class.1'].str.contains('^Non')&df['Amino Acid Change'].str.contains('\
missense=(df['Class.1'].str.contains('^Non')&df['Amino Acid Change'].str.contains('[
nonsense=(df['Class.1'].str.contains('^Non')&df['Amino Acid Change'].str.contains('[
statystyka_nonsense_essential_included(synonymous,nonsense,missense)
statystyka_nonsense_deleterious_excluded(synonymous,nonsense,missense)
#df
```

```
synonymous sides  5058099  synonymous mutations  103 ds 2.0363381578731457e-05
nonsense sides  1166436  nonsense mutations  63 dnonsense 5.40106786827567e-05
P-value nonsense mutation rate=synonymous mutation rate 4.271399249353949e-10
missense sides  17554023  missense mutations  494 dn 2.814169720525033e-05
P-value missense mutation rate=synonymous mutation rate 0.003171810061450255
```

```
dNONSENSE/dS  2.6523432993647864
dN/dS  1.3819756358464028
The corrected dNONSENSE/dS calculations
synonymous sides  5058099  synonymous mutations  103 ds 2.0363381578731457e-05
nonsense sides  233292  nonsense mutations  63 dnonsense 0.00027004783704541945
P-value nonsense mutation rate=synonymous mutation rate 1.2455217870628375e-96
dNONSENSE/dS  13.261443636034942
```

```
C:\Users\Szymon\anaconda3\lib\site-packages\openpyxl\worksheet\_reader.py:312: UserWa
rning: Unknown extension is not supported and will be removed
  warn(msg)
```

In [12]:

```
##The calculation of statistics for the study:"Molecular signatures of aneuploidy-dr
#https://www.nature.com/articles/s41467-019-13669-2
#The authors presented mutations for all experiments together.
#The Excel sheet was imported from SOM. The number of substitutions was calculated u


df=pd.read_excel('Molecular_signatures_of_aneuploidy-driven_adaptive_evolution.xlsx'
nonsense=df['mut_class'].str.contains('Nonsense').value_counts()[True]
missense=df['mut_class'].str.contains('nonSynony').value_counts()[True]
synonymous=df['mut_class'].str.contains('^Synony').value_counts()[True]
statystyka_nonsense_essential_included(synonymous,nonsense,missense)
statystyka_nonsense_deleterious_excluded(synonymous,nonsense,missense)
```

```
synonymous sides  5058099  synonymous mutations  38 ds 7.512703883415489e-06
nonsense sides  1166436  nonsense mutations  7 dnonsense 6.0011865203063e-06
P-value nonsense mutation rate=synonymous mutation rate 0.7216168775354395
missense sides  17554023  missense mutations  87 dn 4.956128860033965e-06
P-value missense mutation rate=synonymous mutation rate 0.040622014247832364
dNONSENSE/dS  0.7988051457151257
dN/dS  0.6596997481791825
The corrected dNONSENSE/dS calculations
synonymous sides  5058099  synonymous mutations  38 ds 7.512703883415489e-06
nonsense sides  233292  nonsense mutations  7 dnonsense 3.000531522726883e-05
P-value nonsense mutation rate=synonymous mutation rate 0.0010410471982000545
dNONSENSE/dS  3.993943551203506
```

In [13]:

```
##The calculation of statistics for the study:"Hidden Complexity of Yeast Adaptation
#https://www.sciencedirect.com/science/article/pii/S0960982218300113#mmc5
#Hidden Complexity of Yeast Adaptation under Simple Evolutionary Conditions
#The Excel sheet was imported from SOM. The number of substitutions was calculated u
df=pd.read_excel('1-s2.0-S0960982218300113-mmc5.xlsx')
missense=df['type'].str.contains('missense_variant').value_counts()[True]
synonymous=df['type'].str.contains('synonymous_variant').value_counts()[True]
nonsense=df['type'].str.contains('stop_gained').value_counts()[True]
#statystyka_nonsense_essential_excluded(synonymous,nonsense,missense)
statystyka_nonsense_essential_included(synonymous,nonsense,missense)
statystyka_nonsense_deleterious_excluded(synonymous,nonsense,missense)
```

```
synonymous sides  5058099  synonymous mutations  65 ds 1.2850677695315968e-05
nonsense sides  1166436  nonsense mutations  45 dnonsense 3.8579056201969075e-05
P-value nonsense mutation rate=synonymous mutation rate 5.338037747858908e-09
missense sides  17554023  missense mutations  221 dn 1.2589706644454095e-05
P-value missense mutation rate=synonymous mutation rate 0.940645459182848
dNONSENSE/dS  3.0021028553249782
dN/dS  0.9796920398247171
The corrected dNONSENSE/dS calculations
synonymous sides  5058099  synonymous mutations  65 ds 1.2850677695315968e-05
nonsense sides  233292  nonsense mutations  45 dnonsense 0.00019289131217529963
P-value nonsense mutation rate=synonymous mutation rate 1.0248054985403515e-75
dNONSENSE/dS  15.010205434193399
```

In [14]:

```
#The calculation of statistics for the study:"Thermo-adaptive evolution to generate
#https://www.sciencedirect.com/science/article/pii/S0168160521000362#t0010
#Table 2, there are no synonymous mutations, maybe they didn't mention them
#statystyka_nonsense_essential_excluded(0,1,11)
statystyka_nonsense_essential_included(0,1,11)
statystyka_nonsense_deleterious_excluded(0,1,11)
```

```
synonymous sides  5058099  synonymous mutations  0 ds 0.0
nonsense sides  1166436  nonsense mutations  1 dnonsense 8.573123600437573e-07
P-value nonsense mutation rate=synonymous mutation rate 0.4230800402270193
missense sides  17554023  missense mutations  11 dn 6.26636982303145e-07
P-value missense mutation rate=synonymous mutation rate 0.15602612966042279
dNONSENSE/dS  error ds=0
dN/dS  error ds=0
The corrected dNONSENSE/dS calculations
synonymous sides  5058099  synonymous mutations  0 ds 0.0
nonsense sides  233292  nonsense mutations  1 dnonsense 4.286473603895547e-06
P-value nonsense mutation rate=synonymous mutation rate 0.02636591897111694
dNONSENSE/dS  error ds=0
```

In [75]:

```
#The calculation of statistics for the study:"Experimental Evolution of Yeast for Hi
#https://academic.oup.com/mbe/article/35/8/1823/4976544
#Experimental Evolution of Yeast for High-Temperature Tolerance
#77 SNVs consisting of 63 nonsynonymous mutations and 14 nonsense mutations (see Sup
#statystyka_nonsense_essential_excluded(14,14,63)
statystyka_nonsense_essential_included(14,14,63)
statystyka_nonsense_deleterious_excluded(14,14,63)
```

```
synonymous sides  5058099  synonymous mutations  14 ds 2.767838272837285e-06
nonsense sides  1166436  nonsense mutations  14 dnonsense 1.20023730406126e-05
P-value nonsense mutation rate=synonymous mutation rate 6.420389920476362e-05
missense sides  17554023  missense mutations  63 dn 3.588920898645285e-06
P-value missense mutation rate=synonymous mutation rate 0.4562913815021056
dNONSENSE/dS  4.336370791024969
dN/dS  1.2966512291797727
The corrected dNONSENSE/dS calculations
synonymous sides  5058099  synonymous mutations  14 ds 2.767838272837285e-06
nonsense sides  233292  nonsense mutations  14 dnonsense 6.001063045453766e-05
P-value nonsense mutation rate=synonymous mutation rate 1.4589728535232437e-29
dNONSENSE/dS  21.681407849390464
```

In [16]:

```
#The calculation of statistics for the study: "Hunger Artists: Yeast Adapted to Carb
#https://journals.plos.org/plosgenetics/article?id=10.1371/journal.pgen.1002202
#Hunger Artists: Yeast Adapted to Carbon Limitation Show Trade-Offs under Carbon Suf
#https://journals.plos.org/plosgenetics/article/figure?id=10.1371/journal.pgen.10022
#statystyka_nonsense_essential_excluded(3,1,18)
#statystyka_nonsense_essential_included(3,1,18)
#I calculated using all excell sheets.
#statystyka_nonsense_essential_excluded(7,1,38)
statystyka_nonsense_essential_included(7,1,38)
statystyka_nonsense_deleterious_excluded(7,1,38)
```

```
synonymous sides  5058099  synonymous mutations  7 ds 1.3839191364186426e-06
nonsense sides  1166436  nonsense mutations  1 dnonsense 8.573123600437573e-07
P-value nonsense mutation rate=synonymous mutation rate 1.0
missense sides  17554023  missense mutations  38 dn 2.164745938865411e-06
P-value missense mutation rate=synonymous mutation rate 0.3586489856671904
dNONSENSE/dS  0.6194815415749956
dN/dS  1.5642141812327417
The corrected dNONSENSE/dS calculations
synonymous sides  5058099  synonymous mutations  7 ds 1.3839191364186426e-06
```

```
nonsense sides  233292  nonsense mutations  1 dnonsense 4.286473603895547e-06
P-value nonsense mutation rate=synonymous mutation rate 0.7997608859054574
dNONSENSE/dS  3.097343978484352
```

In [17]:

```
#The calculation of statistics for the study:"Nitrogen starvation reveals the mitoti
#https://www.nature.com/articles/s41467-020-15880-y#MOESM1
#Nitrogen starvation reveals the mitotic potential of mutants in the S/MAPK pathways
statystyka_nonsense_essential_included(5,1,50)
statystyka_nonsense_deleterious_excluded(5,1,50)
```

```
synonymous sides  5058099  synonymous mutations  5 ds 9.88513668870459e-07
nonsense sides  1166436  nonsense mutations  1 dnonsense 8.573123600437573e-07
P-value nonsense mutation rate=synonymous mutation rate 1.0
missense sides  17554023  missense mutations  50 dn 2.84834991955975e-06
P-value missense mutation rate=synonymous mutation rate 0.02771672085501255
dNONSENSE/dS  0.8672741582049937
dN/dS  2.88144717595505
The corrected dNONSENSE/dS calculations
synonymous sides  5058099  synonymous mutations  5 ds 9.88513668870459e-07
nonsense sides  233292  nonsense mutations  1 dnonsense 4.286473603895547e-06
P-value nonsense mutation rate=synonymous mutation rate 0.6396062772450419
dNONSENSE/dS  4.336281569878093
```

In [18]:

```
#The calculation of statistics for the study:"Improved use of a public good selects 
#https://elifesciences.org/articles/367
#Improved use of a public good selects for the evolution of undifferentiated multice
##The Excel sheet was imported from table 5.The number of substitutions was calculat
#Putative causal mutations in each evolved clone. There are no synonymous mutations
df=pd.read_excel('Improved_undifferentiated_multicellularity.xlsx')
missense=df['Amino acid change'].str.contains('^[A-Z][0-9]+[A-Z]$').value_counts()[T
nonsense=df['Amino acid change'].str.contains('[A-Z][0-9]+\*').value_counts()[True]
synonymous=0
#synonymous=df['type'].str.contains('synonymous_variant').value_counts()[True]
#nonsense=df['type'].str.contains('stop_gained').value_counts()[True]
#statystyka_nonsense_essential_excluded(synonymous,nonsense,missense)
statystyka_nonsense_essential_included(synonymous,nonsense,missense)
statystyka_nonsense_deleterious_excluded(synonymous,nonsense,missense)
```

```
synonymous sides  5058099  synonymous mutations  0 ds 0.0
nonsense sides  1166436  nonsense mutations  19 dnonsense 1.6288934840831388e-05
P-value nonsense mutation rate=synonymous mutation rate 1.5925533257780163e-18
missense sides  17554023  missense mutations  43 dn 2.449580930821385e-06
P-value missense mutation rate=synonymous mutation rate 0.0008468881190994021
dNONSENSE/dS  error ds=0
dN/dS  error ds=0
The corrected dNONSENSE/dS calculations
synonymous sides  5058099  synonymous mutations  0 ds 0.0
nonsense sides  233292  nonsense mutations  19 dnonsense 8.144299847401539e-05
P-value nonsense mutation rate=synonymous mutation rate 1.038641574090113e-86
dNONSENSE/dS  error ds=0
```

In [19]:

```
#The calculation of statistics for the study:"Evolutionary engineering reveals amino
#https://academic.oup.com/femsyr/article/21/4/foab033/6286924?login=true
#Evolutionary engineering reveals amino acid substitutions in Ato2 and Ato3 that all
#See table 3. Authors don't show synonymous mutations, 14 missense, 2 nonsense
statystyka_nonsense_essential_included(0,2,14)
statystyka_nonsense_deleterious_excluded(0,2,14)
```

```
synonymous sides  5058099  synonymous mutations  0 ds 0.0
nonsense sides  1166436  nonsense mutations  2 dnonsense 1.7146247200875146e-06
P-value nonsense mutation rate=synonymous mutation rate 0.041457000496585586
missense sides  17554023  missense mutations  14 dn 7.975379774767299e-07
```

```
P-value missense mutation rate=synonymous mutation rate 0.09144713233388277
dNONSENSE/dS  error ds=0
dN/dS  error ds=0
The corrected dNONSENSE/dS calculations
synonymous sides  5058099  synonymous mutations  0 ds 0.0
nonsense sides  233292  nonsense mutations  2 dnonsense 8.572947207791094e-06
P-value nonsense mutation rate=synonymous mutation rate 1.1571721901605702e-06
dNONSENSE/dS  error ds=0
```

In [20]:

```
#The calculation of statistics for the study: "Long-Term Adaptation to Galactose as
#https://link.springer.com/article/10.1007/s00239-022-10079-9
#The authors claim:
#Of the 1,529 unique mutations, 91% are single nucleotide polymorphisms, most of whi
statystyka_nonsense_essential_included(225,76,712)
statystyka_nonsense_deleterious_excluded(225,76,712)
```

```
synonymous sides  5058099  synonymous mutations  225 ds 4.4483115099170653e-05
nonsense sides  1166436  nonsense mutations  76 dnonsense 6.515573936332555e-05
P-value nonsense mutation rate=synonymous mutation rate 0.004797731835775804
missense sides  17554023  missense mutations  712 dn 4.056050285453084e-05
P-value missense mutation rate=synonymous mutation rate 0.2426952434308508
dNONSENSE/dS  1.4647296894128783
dN/dS  0.9118179507911094
The corrected dNONSENSE/dS calculations
synonymous sides  5058099  synonymous mutations  225 ds 4.4483115099170653e-05
nonsense sides  233292  nonsense mutations  76 dnonsense 0.00032577199389606157
P-value nonsense mutation rate=synonymous mutation rate 2.4659257335799822e-68
dNONSENSE/dS  7.323497762460779
```

In [22]:

```
#The calculation of statistics for the study:"Genomic and transcriptomic analysis of
#https://academic.oup.com/femsyr/article/19/3/foz021/5369625
#The authors claim:
#Genomic and transcriptomic analysis of a coniferyl aldehyde-resistant Saccharomyces
#The SNPs were distributed along all chromosomes, and 66 of 91 intragenic SNPs were
statystyka_nonsense_essential_included(25,4,62)
statystyka_nonsense_deleterious_excluded(25,4,62)
```

```
synonymous sides  5058099  synonymous mutations  25 ds 4.942568344352295e-06
nonsense sides  1166436  nonsense mutations  4 dnonsense 3.429249440175029e-06
P-value nonsense mutation rate=synonymous mutation rate 0.6565736309212007
missense sides  17554023  missense mutations  62 dn 3.53195390025409e-06
P-value missense mutation rate=synonymous mutation rate 0.1948341576736868
dNONSENSE/dS  0.693819326563995
dN/dS  0.7145988996368525
The corrected dNONSENSE/dS calculations
synonymous sides  5058099  synonymous mutations  25 ds 4.942568344352295e-06
nonsense sides  233292  nonsense mutations  4 dnonsense 1.714589441558219e-05
P-value nonsense mutation rate=synonymous mutation rate 0.04450092806554019
dNONSENSE/dS  3.4690252559024746
```

In [23]:

```
#The calculation of statistics for the study: "Induced Mutations in Yeast Cell Popul
#https://journals.plos.org/plosone/article?id=10.1371/journal.pone.0111133
#The authors wrote:
#Finally, we found 18 SNPs in the 28 sequenced genomes: four intergenic, four synony
statystyka_nonsense_essential_included(4,3,7)
statystyka_nonsense_deleterious_excluded(4,3,7)
```

```
synonymous sides  5058099  synonymous mutations  4 ds 7.908109350963672e-07
nonsense sides  1166436  nonsense mutations  3 dnonsense 2.5719370801312716e-06
P-value nonsense mutation rate=synonymous mutation rate 0.24977092991563107
missense sides  17554023  missense mutations  7 dn 3.9876898873836497e-07
P-value missense mutation rate=synonymous mutation rate 0.45201657607295853
```

```
dNONSENSE/dS  3.252278093268726
dN/dS  0.5042532557921338
The corrected dNONSENSE/dS calculations
synonymous sides  5058099  synonymous mutations  4 ds 7.908109350963672e-07
nonsense sides  233292  nonsense mutations  3 dnonsense 1.2859420811686642e-05
P-value nonsense mutation rate=synonymous mutation rate 5.4714672564862376e-05
dNONSENSE/dS  16.261055887042847
```

In [24]:

```
#The calculation of statistics for the study: "Development of a Comprehensive Genotyp
#https://www.sciencedirect.com/science/article/pii/S0092867416310108
#See table 1
#Neutral Diploid
statystyka_nonsense_essential_included(1,0,1)
statystyka_nonsense_deleterious_excluded(1,0,1)
#Neutral Haploid
statystyka_nonsense_essential_included(6,0,25)
statystyka_nonsense_deleterious_excluded(6,0,25)
#Adaptive diploid
statystyka_nonsense_essential_included(31,8,77)
statystyka_nonsense_deleterious_excluded(31,8,77)
#Adaptive haploid
statystyka_nonsense_essential_included(16,31,72)
statystyka_nonsense_deleterious_excluded(16,31,72)
```

```
synonymous sides  5058099  synonymous mutations  1 ds 1.977027337740918e-07
nonsense sides  1166436  nonsense mutations  0 dnonsense 0.0
P-value nonsense mutation rate=synonymous mutation rate 1.0
missense sides  17554023  missense mutations  1 dn 5.6966998391194996e-08
P-value missense mutation rate=synonymous mutation rate 0.9288519731978127
dNONSENSE/dS  0.0
dN/dS  0.28814471759550503
The corrected dNONSENSE/dS calculations
synonymous sides  5058099  synonymous mutations  1 ds 1.977027337740918e-07
nonsense sides  233292  nonsense mutations  0 dnonsense 0.0
P-value nonsense mutation rate=synonymous mutation rate 1.0
dNONSENSE/dS  0.0
synonymous sides  5058099  synonymous mutations  6 ds 1.186216402644551e-06
nonsense sides  1166436  nonsense mutations  0 dnonsense 0.0
P-value nonsense mutation rate=synonymous mutation rate 0.513631773583591
missense sides  17554023  missense mutations  25 dn 1.424174959779875e-06
P-value missense mutation rate=synonymous mutation rate 0.8514894635406378
dNONSENSE/dS  0.0
dN/dS  1.200602989981271
The corrected dNONSENSE/dS calculations
synonymous sides  5058099  synonymous mutations  6 ds 1.186216402644551e-06
nonsense sides  233292  nonsense mutations  0 dnonsense 0.0
P-value nonsense mutation rate=synonymous mutation rate 1.0
dNONSENSE/dS  0.0
synonymous sides  5058099  synonymous mutations  31 ds 6.128784746996846e-06
nonsense sides  1166436  nonsense mutations  8 dnonsense 6.858498880350058e-06
P-value nonsense mutation rate=synonymous mutation rate 0.9373139219779112
missense sides  17554023  missense mutations  77 dn 4.386458876122015e-06
P-value missense mutation rate=synonymous mutation rate 0.1431039380343978
dNONSENSE/dS  1.1190634299419273
dN/dS  0.7157142985436737
The corrected dNONSENSE/dS calculations
synonymous sides  5058099  synonymous mutations  31 ds 6.128784746996846e-06
nonsense sides  233292  nonsense mutations  8 dnonsense 3.429178883116438e-05
P-value nonsense mutation rate=synonymous mutation rate 6.521702357052719e-06
dNONSENSE/dS  5.595202025649152
synonymous sides  5058099  synonymous mutations  16 ds 3.163243740385469e-06
nonsense sides  1166436  nonsense mutations  31 dnonsense 2.6576683161356472e-05
P-value nonsense mutation rate=synonymous mutation rate 5.126124350670797e-16
```

```
missense sides  17554023  missense mutations  72 dn 4.10162388416604e-06
P-value missense mutation rate=synonymous mutation rate 0.41525796875891485
dNONSENSE/dS  8.401718407610876
dN/dS  1.2966512291797727
The corrected dNONSENSE/dS calculations
synonymous sides  5058099  synonymous mutations  16 ds 3.163243740385469e-06
nonsense sides  233292  nonsense mutations  31 dnonsense 0.00013288068172076197
P-value nonsense mutation rate=synonymous mutation rate 1.0341049768490709e-90
dNONSENSE/dS  42.007727708194025
```

In [25]:

```
#https://www.sciencedirect.com/science/article/pii/S1389172316301517
#Isolation and characterization of sake yeast mutants with enhanced isoamyl acetate p
#Tą publikacje trzeba dokładnie przeczytać
```

In [25]:

```
#The calculation os statistics for the study: "Enhanced Wort Fermentation with De No
#https://journals.asm.org/doi/full/10.1128/AEM.02302-17#T2
#Enhanced Wort Fermentation with De Novo Lager Hybrids Adapted to High-Ethanol Envir
#See Supplemental Material 2
#synononymous 9, nonsynonymous 16
statystyka_nonsense_essential_included(9,0,16)
statystyka_nonsense_deleterious_excluded(9,0,16)
```

```
synonymous sides  5058099  synonymous mutations  9 ds 1.7793246039668263e-06
nonsense sides  1166436  nonsense mutations  0 dnonsense 0.0
P-value nonsense mutation rate=synonymous mutation rate 0.3107995146559177
missense sides  17554023  missense mutations  16 dn 9.114719742591199e-07
P-value missense mutation rate=synonymous mutation rate 0.16284845734827286
dNONSENSE/dS  0.0
dN/dS  0.5122572757253422
The corrected dNONSENSE/dS calculations
synonymous sides  5058099  synonymous mutations  9 ds 1.7793246039668263e-06
nonsense sides  233292  nonsense mutations  0 dnonsense 0.0
P-value nonsense mutation rate=synonymous mutation rate 1.0
dNONSENSE/dS  0.0
```

In [26]:

```
#The calculation of statistics for the study:"Genomics of Adaptation Depends on the
#https://academic.oup.com/mbe/article/34/10/2613/3896418?login=true
#Genomics of Adaptation Depends on the Rate of Environmental Change in Experimental
df=pd.read_csv('msx185.txt')
a=df['Amino'].str.extract('p\.([A-Z][a-z][a-z])[0-9]+([A-Z][a-z][a-z])')
df['1']=a[1]
df['0']=a[0]
df1=df.dropna()
#synonymous=sum(a[1]==a[0])
nonsense=df['Amino'].str.contains('[0-9]+\*').value_counts()[True]
lista1=df['GENE'][df['1']==df['1']].values.tolist()
#lista2=df['GENE'][df['0']==df['1']].values.tolist()
#lista2=df['GENE'][df['Amino'].str.contains('\*')].values.tolist()
missense=len(lista1)
synonymous=27 #manually counted
statystyka_nonsense_essential_included(synonymous,nonsense,missense)
statystyka_nonsense_deleterious_excluded(synonymous,nonsense,missense)
```

```
synonymous sides  5058099  synonymous mutations  27 ds 5.3379738119004785e-06
nonsense sides  1166436  nonsense mutations  12 dnonsense 1.0287748320525086e-05
P-value nonsense mutation rate=synonymous mutation rate 0.08542814455225899
missense sides  17554023  missense mutations  90 dn 5.12702985520755e-06
P-value missense mutation rate=synonymous mutation rate 0.9419377035607
dNONSENSE/dS  1.9272759071222083
dN/dS  0.9604823919850168
The corrected dNONSENSE/dS calculations
```

```
synonymous sides  5058099  synonymous mutations  27 ds 5.3379738119004785e-06
nonsense sides  233292  nonsense mutations  12 dnonsense 5.143768324674657e-05
P-value nonsense mutation rate=synonymous mutation rate 2.3729667729718145e-14
dNONSENSE/dS  9.636181266395763
```

In [27]:

```
#The calculation of statistics for the study: "Laboratory evolution for forced gluco
#https://academic.oup.com/femsyr/article/18/6/foy056/4996351?login=true
#Laboratory evolution for forced glucose-xylose co-consumption enables identificatio
#I couldn't find information about synonymous mutations
#3 nonsense, 4 missense
statystyka_nonsense_essential_included(0,3,4)
statystyka_nonsense_deleterious_excluded(0,3,4)
```

```
synonymous sides  5058099  synonymous mutations  0 ds 0.0
nonsense sides  1166436  nonsense mutations  3 dnonsense 2.5719370801312716e-06
P-value nonsense mutation rate=synonymous mutation rate 0.004143187245118398
missense sides  17554023  missense mutations  4 dn 2.2786799356477998e-07
P-value missense mutation rate=synonymous mutation rate 0.6357467590259751
dNONSENSE/dS  error ds=0
dN/dS  error ds=0
The corrected dNONSENSE/dS calculations
synonymous sides  5058099  synonymous mutations  0 ds 0.0
nonsense sides  233292  nonsense mutations  3 dnonsense 1.2859420811686642e-05
P-value nonsense mutation rate=synonymous mutation rate 2.760812160641767e-11
dNONSENSE/dS  error ds=0
```

In [28]:

```
#The calculation of statistics for the study:
#https://journals.plos.org/plosgenetics/article?id=10.1371/journal.pgen.1004041#pgen
#Molecular Specificity, Convergence and Constraint Shape Adaptive Evolution in Nutri
df=pd.read_excel('Molecular_Specificity.xlsx')
nonsense=df['amino acid change'].str.contains('[0-9]\*').value_counts()[True]
missense=df['type'].str.contains('^nSNP').value_counts()[True]-nonsense
synonymous=df['type'].str.contains('^sSNP').value_counts()[True]
statystyka_nonsense_essential_included(synonymous,nonsense,missense)
statystyka_nonsense_deleterious_excluded(synonymous,nonsense,missense)
```

```
synonymous sides  5058099  synonymous mutations  8 ds 1.5816218701927345e-06
nonsense sides  1166436  nonsense mutations  7 dnonsense 6.0011865203063e-06
P-value nonsense mutation rate=synonymous mutation rate 0.014649242368574277
missense sides  17554023  missense mutations  45 dn 2.563514927603775e-06
P-value missense mutation rate=synonymous mutation rate 0.26869341725348156
dNONSENSE/dS  3.794324442146847
dN/dS  1.620814036474716
The corrected dNONSENSE/dS calculations
synonymous sides  5058099  synonymous mutations  8 ds 1.5816218701927345e-06
nonsense sides  233292  nonsense mutations  7 dnonsense 3.000531522726883e-05
P-value nonsense mutation rate=synonymous mutation rate 2.0848011682350095e-13
dNONSENSE/dS  18.971231868216655
```

In [29]:

```
#It should be excluded from the analysis
#The calculation of statistics for the study:"Phenotypic and molecular evolution acr
#https://elifesciences.org/articles/63910.pdf
#They wrote "The relative prevalence of different types of fixed mutations across st
#environments are similar, with roughly 45–50% missense mutations, 40–45% synonymous
#I couldn't find better data and information in their paper. I assumed all indels ar

statystyka_nonsense_essential_included(45,5,45)
statystyka_nonsense_deleterious_excluded(45,5,45)
statystyka_nonsense_essential_included(40,10,45)
statystyka_nonsense_deleterious_excluded(40,10,45)
```

```
synonymous sides  5058099  synonymous mutations  45 ds 8.896623019834132e-06
nonsense sides  1166436  nonsense mutations  5 dnonsense 4.286561800218786e-06
P-value nonsense mutation rate=synonymous mutation rate 0.1607981957225066
missense sides  17554023  missense mutations  45 dn 2.563514927603775e-06
P-value missense mutation rate=synonymous mutation rate 7.097861383701274e-10
dNONSENSE/dS  0.481818976780552
dN/dS  0.28814471759550503
The corrected dNONSENSE/dS calculations
synonymous sides  5058099  synonymous mutations  45 ds 8.896623019834132e-06
nonsense sides  233292  nonsense mutations  5 dnonsense 2.1432368019477737e-05
P-value nonsense mutation rate=synonymous mutation rate 0.11380232784251652
dNONSENSE/dS  2.4090453165989403
synonymous sides  5058099  synonymous mutations  40 ds 7.908109350963673e-06
nonsense sides  1166436  nonsense mutations  10 dnonsense 8.573123600437572e-06
P-value nonsense mutation rate=synonymous mutation rate 0.962327332734382
missense sides  17554023  missense mutations  45 dn 2.563514927603775e-06
P-value missense mutation rate=synonymous mutation rate 9.698375397912629e-08
dNONSENSE/dS  1.084092697756242
dN/dS  0.3241628072949431
The corrected dNONSENSE/dS calculations
synonymous sides  5058099  synonymous mutations  40 ds 7.908109350963673e-06
nonsense sides  233292  nonsense mutations  10 dnonsense 4.2864736038955474e-05
P-value nonsense mutation rate=synonymous mutation rate 5.017941290037356e-07
dNONSENSE/dS  5.420351962347616
```

In [30]:

```
# The calculation of statistics for the study: "Altered access to beneficial mutatio
#https://www.nature.com/articles/s41559-018-0503-9
#Authors wrote:
#"Of the 850 mutations, 342 are intergenic, 114 are synonymous, 336 are missense, 23
statystyka_nonsense_essential_included(114,23,336)
statystyka_nonsense_deleterious_excluded(114,23,336)
```

```
synonymous sides  5058099  synonymous mutations  114 ds 2.2538111650246465e-05
nonsense sides  1166436  nonsense mutations  23 dnonsense 1.9718184281006416e-05
P-value nonsense mutation rate=synonymous mutation rate 0.6342738818083382
missense sides  17554023  missense mutations  336 dn 1.914091145944152e-05
P-value missense mutation rate=synonymous mutation rate 0.14637712941198347
dNONSENSE/dS  0.8748818262594235
dN/dS  0.8492686413341202
The corrected dNONSENSE/dS calculations
synonymous sides  5058099  synonymous mutations  114 ds 2.2538111650246465e-05
nonsense sides  233292  nonsense mutations  23 dnonsense 9.858889288959758e-05
P-value nonsense mutation rate=synonymous mutation rate 7.40193308043399e-12
dNONSENSE/dS  4.374319127508603
```

In [31]:

```
#The calculation of statistics for the study:"Evolutionary adaptation after cripplin
#https://www.ncbi.nlm.nih.gov/pmc/articles/PMC4630673/
#Evolutionary adaptation after crippling cell polarization follows reproducible traj
#I used data from table SOM-1 (counted manually) Nonsense 17,Missense 8, Synonymous
statystyka_nonsense_essential_included(0,17,8)
statystyka_nonsense_deleterious_excluded(0,17,8)
```

```
synonymous sides  5058099  synonymous mutations  0 ds 0.0
nonsense sides  1166436  nonsense mutations  17 dnonsense 1.4574310120743873e-05
P-value nonsense mutation rate=synonymous mutation rate 1.283217720873529e-16
missense sides  17554023  missense mutations  8 dn 4.5573598712955997e-07
P-value missense mutation rate=synonymous mutation rate 0.2739282113059329
dNONSENSE/dS  error ds=0
dN/dS  error ds=0
The corrected dNONSENSE/dS calculations
synonymous sides  5058099  synonymous mutations  0 ds 0.0
nonsense sides  233292  nonsense mutations  17 dnonsense 7.28700512662243e-05
```

```
P-value nonsense mutation rate=synonymous mutation rate 2.8100699034257123e-77
dNONSENSE/dS  error ds=0
```

In [32]:

```
#The calculation of statistics for the study: "The Dynamics of Diverse Segmental Amp
#https://academic.oup.com/g3journal/article/4/3/399/6025590
#
#The data from table S2 is used (counted manually)
#counted manually
statystyka_nonsense_essential_included(1,2,10)
statystyka_nonsense_deleterious_excluded(1,2,10)
```

```
synonymous sides  5058099  synonymous mutations  1 ds 1.977027337740918e-07
nonsense sides  1166436  nonsense mutations  2 dnonsense 1.7146247200875146e-06
P-value nonsense mutation rate=synonymous mutation rate 0.1652812486540442
missense sides  17554023  missense mutations  10 dn 5.6966998391195e-07
P-value missense mutation rate=synonymous mutation rate 0.4870406353790627
dNONSENSE/dS  8.672741582049937
dN/dS  2.88144717595505
The corrected dNONSENSE/dS calculations
synonymous sides  5058099  synonymous mutations  1 ds 1.977027337740918e-07
nonsense sides  233292  nonsense mutations  2 dnonsense 8.572947207791094e-06
P-value nonsense mutation rate=synonymous mutation rate 0.00011981980094534615
dNONSENSE/dS  43.36281569878093
```

In [34]:

```
#https://www.nature.com/articles/s41467-021-25440-7#MOESM1
#Changes in the distribution of fitness effects and adaptive mutational spectra foll
#Specifically, 53 out of 95 causative mutations (56%) from the 1st step evolution re
#There is no usefull data
```

In [33]:

```
#The calculation of statistics for the study:"Exploring a Local Genetic Interaction 
#https://academic.oup.com/mbe/article/38/8/3144/6179813
#The pandas package was applied for calculations.
df=pd.read_excel('Supplemental_Dataset_1Exploring_Experiments.xlsx')
synonymous=df['Type'].str.contains('synonymous').value_counts()[True]
nonsense=df['Type'].str.contains('nonsense').value_counts()[True]
missense=df['Type'].str.contains('missense').value_counts()[True]
print(synonymous,nonsense, missense)
statystyka_nonsense_essential_included(synonymous,nonsense,missense)
statystyka_nonsense_deleterious_excluded(synonymous,nonsense,missense)
print(synonymous,nonsense,missense)
```

```
90 17 342
synonymous sides  5058099  synonymous mutations  90 ds 1.7793246039668264e-05
nonsense sides  1166436  nonsense mutations  17 dnonsense 1.4574310120743873e-05
P-value nonsense mutation rate=synonymous mutation rate 0.5273933240435861
missense sides  17554023  missense mutations  342 dn 1.948271344978869e-05
P-value missense mutation rate=synonymous mutation rate 0.4788236415551771
dNONSENSE/dS  0.8190922605269384
dN/dS  1.094949926862919
The corrected dNONSENSE/dS calculations
synonymous sides  5058099  synonymous mutations  90 ds 1.7793246039668264e-05
nonsense sides  233292  nonsense mutations  17 dnonsense 7.28700512662243e-05
P-value nonsense mutation rate=synonymous mutation rate 2.885558936359771e-08
dNONSENSE/dS  4.095377038218198
90 17 342
```

In [34]:

```
#The calculations for the study: "PHENOTYPIC AND GENOTYPIC CONVERGENCES ARE INFLUENC
#https://onlinelibrary.wiley.com/doi/full/10.1111/evo.12302
#
#Table 4 manually counted
#synonymous 3
```

```
#nonsense 10
#disruptive missense 6
#Tolerated missense 4
statystyka_nonsense_essential_included(3,10,10)
statystyka_nonsense_deleterious_excluded(3,10,10)
```

```
synonymous sides  5058099  synonymous mutations  3 ds 5.931082013222755e-07
nonsense sides  1166436  nonsense mutations  10 dnonsense 8.573123600437572e-06
P-value nonsense mutation rate=synonymous mutation rate 5.151757051097131e-07
missense sides  17554023  missense mutations  10 dn 5.6966998391195e-07
P-value missense mutation rate=synonymous mutation rate 1.0
dNONSENSE/dS  14.45456930341656
dN/dS  0.9604823919850166
The corrected dNONSENSE/dS calculations
synonymous sides  5058099  synonymous mutations  3 ds 5.931082013222755e-07
nonsense sides  233292  nonsense mutations  10 dnonsense 4.2864736038955474e-05
P-value nonsense mutation rate=synonymous mutation rate 1.7200112476863895e-33
dNONSENSE/dS  72.2713594979682
```

In [35]:

```
#The calculations for the study:"Rescuing yeast from cell death enables overproducti
#Rescuing yeast from cell death enables overproduction of fatty acids from sole meth
#I used data from sumplementary metherials (table 6).
#https://static-content.springer.com/esm/art%3A10.1038%2Fs42255-022-00601-0/MediaObj
statystyka_nonsense_essential_included(0,1,13)
statystyka_nonsense_deleterious_excluded(0,1,13)
```

```
synonymous sides  5058099  synonymous mutations  0 ds 0.0
nonsense sides  1166436  nonsense mutations  1 dnonsense 8.573123600437573e-07
P-value nonsense mutation rate=synonymous mutation rate 0.4230800402270193
missense sides  17554023  missense mutations  13 dn 7.40570979085535e-07
P-value missense mutation rate=synonymous mutation rate 0.10901254772956065
dNONSENSE/dS  error ds=0
dN/dS  error ds=0
The corrected dNONSENSE/dS calculations
synonymous sides  5058099  synonymous mutations  0 ds 0.0
nonsense sides  233292  nonsense mutations  1 dnonsense 4.286473603895547e-06
P-value nonsense mutation rate=synonymous mutation rate 0.02636591897111694
dNONSENSE/dS  error ds=0
```

In [ ]:

```
#https://link.springer.com/article/10.1007/s11274-019-2762-2
#Evolutionary engineering and molecular characterization of a caffeine-resistant Sac

#Whole genome re-sequencing of Caf905-2 and the reference strain was performed using
#next-generation sequencing platform and the library prep platform Ion Chef, to dete
#caffeine-resistant strain Caf905-2. This analysis created 21.1 and 22.1 million rea
#respectively. The raw sequence reads were about × 178 and × 192 depth coverage for
#respectively. Following the alignment to the genome sequence of CEN.PK113-7D (Nijkar
#a total of three single nucleotide polymorphisms (SNPs) were detected in Caf905-2,
#All of these SNPs were missense SNPs: a transversioFEn SNP (A2456T) was found in th
#Also, PDR5, encoding an ABC transporter, conferred a transversion SNP (G2008T) corr
#The third missense SNP was found in RIM8 gene as a transition SNP (A1097G) which co
#3 missense,0 nonsense, 0 synonymous
```

In [36]:

```
#The calculations for the study: "Disadvantages and benefits of evolved unicellulari
#https://onlinelibrary.wiley.com/doi/full/10.1002/ece3.5322
#Manual counting table 1 (probably they counted synonymous mutations)
statystyka_nonsense_essential_included(0,1,8)
statystyka_nonsense_deleterious_excluded(0,1,8)
```

```
synonymous sides  5058099  synonymous mutations  0 ds 0.0
nonsense sides  1166436  nonsense mutations  1 dnonsense 8.573123600437573e-07
P-value nonsense mutation rate=synonymous mutation rate 0.4230800402270193
```

```
missense sides  17554023  missense mutations  8 dn 4.557359871295597e-07
P-value missense mutation rate=synonymous mutation rate 0.2739282113059329
dNONSENSE/dS  error ds=0
dN/dS  error ds=0
The corrected dNONSENSE/dS calculations
synonymous sides  5058099  synonymous mutations  0 ds 0.0
nonsense sides  233292  nonsense mutations  1 dnonsense 4.286473603895547e-06
P-value nonsense mutation rate=synonymous mutation rate 0.02636591897111694
dNONSENSE/dS  error ds=0
```

In [37]:

```
#The calculations for the study:Reconstruction of thermotolerant yeast by one-point 
#https://www.nature.com/articles/srep23157#MOESM59
statystyka_nonsense_essential_included(5,0,26)
statystyka_nonsense_deleterious_excluded(5,0,26)
```

```
synonymous sides  5058099  synonymous mutations  5 ds 9.88513668870459e-07
nonsense sides  1166436  nonsense mutations  0 dnonsense 0.0
P-value nonsense mutation rate=synonymous mutation rate 0.6165272649833601
missense sides  17554023  missense mutations  26 dn 1.48114195817107e-06
P-value missense mutation rate=synonymous mutation rate 0.5364307938744409
dNONSENSE/dS  0.0
dN/dS  1.4983525314966262
The corrected dNONSENSE/dS calculations
synonymous sides  5058099  synonymous mutations  5 ds 9.88513668870459e-07
nonsense sides  233292  nonsense mutations  0 dnonsense 0.0
P-value nonsense mutation rate=synonymous mutation rate 1.0
dNONSENSE/dS  0.0
```

In [38]:

```
#The calculations for the study: "Rapid Chagas Disease Drug Target Discovery Using D
#https://pubs.acs.org/doi/full/10.1021/acschembio.6b01037
#table 1 manually counted
#nonsense 0
#missense 9
#synonymous 8
statystyka_nonsense_essential_included(8,0,9)
statystyka_nonsense_deleterious_excluded(8,0,9)
```

```
synonymous sides  5058099  synonymous mutations  8 ds 1.5816218701927345e-06
nonsense sides  1166436  nonsense mutations  0 dnonsense 0.0
P-value nonsense mutation rate=synonymous mutation rate 0.3653352063111819
missense sides  17554023  missense mutations  9 dn 5.12702985520755e-07
P-value missense mutation rate=synonymous mutation rate 0.031407498811091956
dNONSENSE/dS  0.0
dN/dS  0.3241628072949432
The corrected dNONSENSE/dS calculations
synonymous sides  5058099  synonymous mutations  8 ds 1.5816218701927345e-06
nonsense sides  233292  nonsense mutations  0 dnonsense 0.0
P-value nonsense mutation rate=synonymous mutation rate 1.0
dNONSENSE/dS  0.0
```

In [39]:

```
#The calculations for the study:"Elucidating aromatic acid tolerance at low pH in Sa
#https://www.pnas.org/doi/10.1073/pnas.2013044117#supplementary-materials
#The SOM file was covered from PDF to Excel format. Tables were copies as text. The

#echo "cumaric acid"
#echo "nonsense"
#awk '  {gsub(/[ ",]/,"\n",$0);print $0}' table1 |awk '$0~/^[A-Z][0-9]+\*/' |wc
#echo "nonredundant nonsense"
#awk '  {gsub(/[ ",]/,"\n",$0);print $0}' table1 |awk '$0~/^[A-Z][0-9]+\*/' |sort -u
#echo "synonymous"
#awk ' {gsub(/[ ",]/,"\n",$0);print $0}' table1 |awk '$0~/^[A-Z][0-9]+[A-Z]/' |awk '
#echo "nonredundant synonymous"
```

```
#awk ' {gsub(/[ ",]/,"\n",$0);print $0}' table1 |awk '$0~/^[A-Z][0-9]+[A-Z]/' |sort
#echo "missense"
#awk ' {gsub(/[ ",]/,"\n",$0);print $0}' table1 |awk '$0~/^[A-Z][0-9]+[A-Z]/' |awk '
#echo "nonredudndant missense"
#awk ' {gsub(/[ ",]/,"\n",$0);print $0}' table1 |awk '$0~/^[A-Z][0-9]+[A-Z]/' |sort
#echo "ferulic acid"
#echo "nonsense"
#awk '  {gsub(/[ ",]/,"\n",$0);print $0}' table2 |awk '$0~/^[A-Z][0-9]+\*/' |wc
#echo "nonredundant nonsense"
#awk '  {gsub(/[ ",]/,"\n",$0);print $0}' table2 |awk '$0~/^[A-Z][0-9]+\*/' |sort -u
#echo "synonymous"
#awk ' {gsub(/[ ",]/,"\n",$0);print $0}' table2 |awk '$0~/^[A-Z][0-9]+[A-Z]/' |awk '
#echo "nonredundant synonymous"
#awk ' {gsub(/[ ",]/,"\n",$0);print $0}' table2 |awk '$0~/^[A-Z][0-9]+[A-Z]/' |sort
#echo "missense"
#awk ' {gsub(/[ ",]/,"\n",$0);print $0}' table2 |awk '$0~/^[A-Z][0-9]+[A-Z]/' |awk '
#echo "nonredudndant missense"
#awk ' {gsub(/[ ",]/,"\n",$0);print $0}' table2 |awk '$0~/^[A-Z][0-9]+[A-Z]/' |sort


#cumaric acid
statystyka_nonsense_essential_included(2,6,77)
statystyka_nonsense_deleterious_excluded(2,6,77)
#or (redundant mutations are counted only one time)
statystyka_nonsense_essential_included(2,3,39)
statystyka_nonsense_deleterious_excluded(2,3,39)
#ferulic acid
statystyka_nonsense_essential_included(1,23,77)
statystyka_nonsense_deleterious_excluded(1,23,77)
#or (redundant mutations are counted only one time)
statystyka_nonsense_essential_included(1,8,44)
statystyka_nonsense_deleterious_excluded(1,8,44)
```

```
synonymous sides  5058099  synonymous mutations  2 ds 3.954054675481836e-07
nonsense sides  1166436  nonsense mutations  6 dnonsense 5.143874160262543e-06
P-value nonsense mutation rate=synonymous mutation rate 0.0002891343714030534
missense sides  17554023  missense mutations  77 dn 4.386458876122015e-06
P-value missense mutation rate=synonymous mutation rate 4.201163082750945e-05
dNONSENSE/dS  13.009112373074904
dN/dS  11.093571627426943
The corrected dNONSENSE/dS calculations
synonymous sides  5058099  synonymous mutations  2 ds 3.954054675481836e-07
nonsense sides  233292  nonsense mutations  6 dnonsense 2.5718841623373285e-05
P-value nonsense mutation rate=synonymous mutation rate 7.684659203121844e-19
dNONSENSE/dS  65.04422354817139
synonymous sides  5058099  synonymous mutations  2 ds 3.954054675481836e-07
nonsense sides  1166436  nonsense mutations  3 dnonsense 2.5719370801312716e-06
P-value nonsense mutation rate=synonymous mutation rate 0.07324701561413989
missense sides  17554023  missense mutations  39 dn 2.221712937256605e-06
P-value missense mutation rate=synonymous mutation rate 0.012412027139104677
dNONSENSE/dS  6.504556186537452
dN/dS  5.6188219931123475
The corrected dNONSENSE/dS calculations
synonymous sides  5058099  synonymous mutations  2 ds 3.954054675481836e-07
nonsense sides  233292  nonsense mutations  3 dnonsense 1.2859420811686642e-05
P-value nonsense mutation rate=synonymous mutation rate 6.84209409185564e-07
dNONSENSE/dS  32.522111774085694
synonymous sides  5058099  synonymous mutations  1 ds 1.977027337740918e-07
nonsense sides  1166436  nonsense mutations  23 dnonsense 1.9718184281006416e-05
P-value nonsense mutation rate=synonymous mutation rate 4.646041811520093e-21
missense sides  17554023  missense mutations  77 dn 4.386458876122015e-06
P-value missense mutation rate=synonymous mutation rate 1.4693280928235542e-05
dNONSENSE/dS  99.73652819357427
dN/dS  22.187143254853886
```

```
The corrected dNONSENSE/dS calculations
synonymous sides  5058099  synonymous mutations  1 ds 1.977027337740918e-07
nonsense sides  233292  nonsense mutations  23 dnonsense 9.858889288959758e-05
P-value nonsense mutation rate=synonymous mutation rate 7.598731151806819e-101
dNONSENSE/dS  498.67238053598066
synonymous sides  5058099  synonymous mutations  1 ds 1.977027337740918e-07
nonsense sides  1166436  nonsense mutations  8 dnonsense 6.858498880350058e-06
P-value nonsense mutation rate=synonymous mutation rate 6.83951799048147e-07
missense sides  17554023  missense mutations  44 dn 2.50654792921258e-06
P-value missense mutation rate=synonymous mutation rate 0.002181721573748947
dNONSENSE/dS  34.69096632819975
dN/dS  12.678367574202221
The corrected dNONSENSE/dS calculations
synonymous sides  5058099  synonymous mutations  1 ds 1.977027337740918e-07
nonsense sides  233292  nonsense mutations  8 dnonsense 3.429178883116438e-05
P-value nonsense mutation rate=synonymous mutation rate 8.968893131083665e-31
dNONSENSE/dS  173.4512627951237
```

In [40]:

```
#The calculation of statistics for the study: "Evolutionary and reverse engineering
#https://microbialcellfactories.biomedcentral.com/articles/10.1186/s12934-022-01996-
#See FIG2 Missense 6, nonsense 1, synonymous 6, frameshift 5
statystyka_nonsense_essential_included(6,1,6)
statystyka_nonsense_deleterious_excluded(6,1,6)
```

```
synonymous sides  5058099  synonymous mutations  6 ds 1.186216402644551e-06
nonsense sides  1166436  nonsense mutations  1 dnonsense 8.573123600437573e-07
P-value nonsense mutation rate=synonymous mutation rate 1.0
missense sides  17554023  missense mutations  6 dn 3.4180199034717e-07
P-value missense mutation rate=synonymous mutation rate 0.05110987209274868
dNONSENSE/dS  0.722728465170828
dN/dS  0.288144717595505
The corrected dNONSENSE/dS calculations
synonymous sides  5058099  synonymous mutations  6 ds 1.186216402644551e-06
nonsense sides  233292  nonsense mutations  1 dnonsense 4.286473603895547e-06
P-value nonsense mutation rate=synonymous mutation rate 0.7245814364319114
dNONSENSE/dS  3.61356797489841
```

In [41]:

```
#The calculations of statistics for the study:"Adaptive genome duplication affects p
#https://journals.plos.org/plosgenetics/article?id=10.1371/journal.pgen.1007396
df=pd.read_excel('Adaptive_genome_MOESM5_ESM.xlsx')
#synonymous=df['Coding Effect'].str.contains('synonymous').value_counts()[True]
#nonsense=df['Coding Effect'].str.contains('nonsense').value_counts()[True]
#missense=df['Coding Effect'].str.contains('missense').value_counts()[True]
#nonsense=df[df['Coding Effect'].str.contains('nonsense')].value_counts()[True]
#print(synonymous,nonsense, missense)
#statystyka_nonsense_essential_excluded(synonymous,nonsense,missense)
#statystyka_nonsense_essential_included(synonymous,nonsense,missense)
print(synonymous,nonsense,missense)
list1=df['Coding Effect'].tolist()
missense=list1.count('missense')
nonsense=list1.count('nonsense')
synonymous=list1.count('synonymous')
print(synonymous,nonsense, missense)
statystyka_nonsense_essential_included(synonymous,nonsense,missense)
statystyka_nonsense_deleterious_excluded(synonymous,nonsense,missense)
```

```
90 17 342
1179 355 3903
synonymous sides  5058099  synonymous mutations  1179 ds 0.00023309152311965425
nonsense sides  1166436  nonsense mutations  355 dnonsense 0.0003043458878155338
P-value nonsense mutation rate=synonymous mutation rate 1.1565145296296747e-05
missense sides  17554023  missense mutations  3903 dn 0.00022234219472083407
P-value missense mutation rate=synonymous mutation rate 0.1603647413257256
```

```
dNONSENSE/dS  1.3056926470007324
dN/dS  0.9538836579942799
The corrected dNONSENSE/dS calculations
synonymous sides  5058099  synonymous mutations  1179 ds 0.00023309152311965425
nonsense sides  233292  nonsense mutations  355 dnonsense 0.0015216981293829192
P-value nonsense mutation rate=synonymous mutation rate 2.239481638725884e-278
dNONSENSE/dS  6.528328911394075
```

In [42]:

```
#The calculations for the study:"Molecular characterization of clonal interference d
#https://www.nature.com/articles/ng.280#Tab1
#Molecular characterization of clonal interference during adaptive evolution in asex
#There are no data about synonymous mutations
#https://www.nature.com/articles/ng.280/tables/1
statystyka_nonsense_essential_included(0,2,6)
statystyka_nonsense_deleterious_excluded(0,2,6)
```

```
synonymous sides  5058099  synonymous mutations  0 ds 0.0
nonsense sides  1166436  nonsense mutations  2 dnonsense 1.7146247200875146e-06
P-value nonsense mutation rate=synonymous mutation rate 0.041457000496585586
missense sides  17554023  missense mutations  6 dn 3.4180199034717e-07
P-value missense mutation rate=synonymous mutation rate 0.40935750139635696
dNONSENSE/dS  error ds=0
dN/dS  error ds=0
The corrected dNONSENSE/dS calculations
synonymous sides  5058099  synonymous mutations  0 ds 0.0
nonsense sides  233292  nonsense mutations  2 dnonsense 8.572947207791094e-06
P-value nonsense mutation rate=synonymous mutation rate 1.1571721901605702e-06
dNONSENSE/dS  error ds=0
```

In [43]:

```
#The calculations of statistics for the study:"Reciprocal Sign Epistasis between Fre
#https://journals.plos.org/plosgenetics/article?id=10.1371/journal.pgen.1002056
#Figure1
#synonymous 1
#nonsense 4
#missense 6
statystyka_nonsense_essential_included(1,4,6)
statystyka_nonsense_deleterious_excluded(1,4,6)
```

```
synonymous sides  5058099  synonymous mutations  1 ds 1.977027337740918e-07
nonsense sides  1166436  nonsense mutations  4 dnonsense 3.429249440175029e-06
P-value nonsense mutation rate=synonymous mutation rate 0.0033106120608519325
missense sides  17554023  missense mutations  6 dn 3.4180199034717e-07
P-value missense mutation rate=synonymous mutation rate 0.9523896073900209
dNONSENSE/dS  17.345483164099875
dN/dS  1.72886830557303
The corrected dNONSENSE/dS calculations
synonymous sides  5058099  synonymous mutations  1 ds 1.977027337740918e-07
nonsense sides  233292  nonsense mutations  4 dnonsense 1.714589441558219e-05
P-value nonsense mutation rate=synonymous mutation rate 9.05326362484129e-13
dNONSENSE/dS  86.72563139756186
```

In [44]:

```
#The calculations of statistics for the study:"The Valley-of-Death: Reciprocal sign
#https://www.sciencedirect.com/science/article/pii/S0888754314002122
#43 of these resulted in non-synonymous changes to coding sequences, while 4 resulte
statystyka_nonsense_essential_included(4,6,43)
statystyka_nonsense_deleterious_excluded(4,6,43)
```

```
synonymous sides  5058099  synonymous mutations  4 ds 7.908109350963672e-07
nonsense sides  1166436  nonsense mutations  6 dnonsense 5.143874160262543e-06
P-value nonsense mutation rate=synonymous mutation rate 0.003298628559533414
missense sides  17554023  missense mutations  43 dn 2.449580930821385e-06
P-value missense mutation rate=synonymous mutation rate 0.03530006648143626
```

```
dNONSENSE/dS  6.504556186537452
dN/dS  3.0975557141516794
The corrected dNONSENSE/dS calculations
synonymous sides  5058099  synonymous mutations  4 ds 7.908109350963672e-07
nonsense sides  233292  nonsense mutations  6 dnonsense 2.5718841623373285e-05
P-value nonsense mutation rate=synonymous mutation rate 6.552676764854329e-15
dNONSENSE/dS  32.522111774085694
```

In [45]:

```
#The calculation of statistics for the study:Adaptation by Loss of Heterozygosity in
#https://academic.oup.com/genetics/article/213/2/665/5930632?login=true
#It is written in this paper:
#"We identified 139 new single nucleotide mutations in the 54 evolved lines (46 in v
#Using less-stringent quality filtering and inclusion of indels, a total of 334 puta
#Using the conservative set going forward (...) Most of the mutations were likely fu
print(0.22*139,0.08*139,0.54*139)
statystyka_nonsense_essential_included(31,11,75)
statystyka_nonsense_deleterious_excluded(31,11,75)
```

```
30.580000000000002 11.120000000000001 75.06
synonymous sides  5058099  synonymous mutations  31 ds 6.128784746996846e-06
nonsense sides  1166436  nonsense mutations  11 dnonsense 9.43043596048133e-06
P-value nonsense mutation rate=synonymous mutation rate 0.2984593756518609
missense sides  17554023  missense mutations  75 dn 4.272524879339625e-06
P-value missense mutation rate=synonymous mutation rate 0.1135686330993418
dNONSENSE/dS  1.53871221617015
dN/dS  0.6971243167633187
The corrected dNONSENSE/dS calculations
synonymous sides  5058099  synonymous mutations  31 ds 6.128784746996846e-06
nonsense sides  233292  nonsense mutations  11 dnonsense 4.715120964285102e-05
P-value nonsense mutation rate=synonymous mutation rate 8.026317830550793e-11
dNONSENSE/dS  7.693402785267584
```

In [46]:

```
#The calculation of statiscs for the study:"yEvo: experimental evolution in high sch
#https://academic.oup.com/g3journal/article/12/11/jkac246/6730751?login=true
df=pd.read_excel('yEvo.xlsx',sheet_name='Table S6')
nonsense=df['PROTEIN'].str.contains('\*$').value_counts()[True]
missense=df['ANNOTATION'].str.contains('coding-non').value_counts()[True]-nonsense
synonymous=df['ANNOTATION'].str.contains('coding-syn').value_counts()[True]
statystyka_nonsense_essential_included(synonymous,nonsense,missense)
statystyka_nonsense_deleterious_excluded(synonymous,nonsense,missense)
print(synonymous,nonsense,missense)
```

```
synonymous sides  5058099  synonymous mutations  76 ds 1.5025407766830978e-05
nonsense sides  1166436  nonsense mutations  59 dnonsense 5.0581429242581674e-05
P-value nonsense mutation rate=synonymous mutation rate 2.430909482188615e-13
missense sides  17554023  missense mutations  555 dn 3.1616684107113226e-05
P-value missense mutation rate=synonymous mutation rate 6.580710259733278e-10
dNONSENSE/dS  3.3663931140851724
dN/dS  2.1042147140198066
The corrected dNONSENSE/dS calculations
synonymous sides  5058099  synonymous mutations  76 ds 1.5025407766830978e-05
nonsense sides  233292  nonsense mutations  59 dnonsense 0.0002529019426298373
P-value nonsense mutation rate=synonymous mutation rate 1.5743708813746046e-107
dNONSENSE/dS  16.83161925150049
76 59 555
```

In [47]:

```
#The calculation of statistics for the study:"Beneficial mutations for carotenoid pr
#https://academic.oup.com/jimb/article/46/12/1793/5996839?login=true
#See table number 1
statystyka_nonsense_essential_included(1,1,8)
statystyka_nonsense_deleterious_excluded(1,1,8)
```

```
synonymous sides  5058099  synonymous mutations  1 ds 1.977027337740918e-07
nonsense sides  1166436  nonsense mutations  1 dnonsense 8.573123600437573e-07
P-value nonsense mutation rate=synonymous mutation rate 0.8205081794951997
missense sides  17554023  missense mutations  8 dn 4.5573598712955997e-07
P-value missense mutation rate=synonymous mutation rate 0.6814270143071972
dNONSENSE/dS  4.336370791024969
dN/dS  2.3051577407640402
The corrected dNONSENSE/dS calculations
synonymous sides  5058099  synonymous mutations  1 ds 1.977027337740918e-07
nonsense sides  233292  nonsense mutations  1 dnonsense 4.286473603895547e-06
P-value nonsense mutation rate=synonymous mutation rate 0.1560539506567918
dNONSENSE/dS  21.681407849390464
```

In [48]:

```
#https://journals.asm.org/doi/full/10.1128/AEM.00388-20
#Adaptive Laboratory Evolution and Reverse Engineering of Single-Vitamin Prototrophi
#There is no information about synonymous mutations
#82 missense and 4 nonsense
statystyka_nonsense_essential_included(0,4,83)
statystyka_nonsense_deleterious_excluded(0,4,83)
```

```
synonymous sides  5058099  synonymous mutations  0 ds 0.0
nonsense sides  1166436  nonsense mutations  4 dnonsense 3.429249440175029e-06
P-value nonsense mutation rate=synonymous mutation rate 0.00042487147354268413
missense sides  17554023  missense mutations  83 dn 4.7282608664691845e-06
P-value missense mutation rate=synonymous mutation rate 1.9484804643316447e-06
dNONSENSE/dS  error ds=0
dN/dS  error ds=0
The corrected dNONSENSE/dS calculations
synonymous sides  5058099  synonymous mutations  0 ds 0.0
nonsense sides  233292  nonsense mutations  4 dnonsense 1.714589441558219e-05
P-value nonsense mutation rate=synonymous mutation rate 5.734925138889457e-16
dNONSENSE/dS  error ds=0
```

In [49]:

```
#https://link.springer.com/article/10.1186/s12934-021-01598-z#MOESM1
#Adaptive laboratory evolution of β-caryophyllene producing Saccharomyces cerevisiae
df=pd.read_excel('Adaptive-caryophyllene.xlsx')
nonsense=df['Annotation'].str.contains('[0-9]\*').value_counts()[True]
synonymous=df['Annotation'].str.contains(r'^([A-Z])[0-9]+\1',regex=True).value_count
missense=df['Annotation'].str.contains(r'^([A-Z])[0-9]+[A-Z]',regex=True).value_coun
statystyka_nonsense_essential_included(synonymous,nonsense,missense)
statystyka_nonsense_deleterious_excluded(synonymous,nonsense,missense)
```

```
synonymous sides  5058099  synonymous mutations  26 ds 5.140271078126387e-06
nonsense sides  1166436  nonsense mutations  7 dnonsense 6.0011865203063e-06
P-value nonsense mutation rate=synonymous mutation rate 0.8878907138666431
missense sides  17554023  missense mutations  21 dn 1.196306966215095e-06
P-value missense mutation rate=synonymous mutation rate 1.5560200655854413e-07
dNONSENSE/dS  1.1674844437374916
dN/dS  0.2327322719040618
The corrected dNONSENSE/dS calculations
synonymous sides  5058099  synonymous mutations  26 ds 5.140271078126387e-06
nonsense sides  233292  nonsense mutations  7 dnonsense 3.000531522726883e-05
P-value nonsense mutation rate=synonymous mutation rate 1.8867481713369742e-05
dNONSENSE/dS  5.837302113297433
```

```
C:\Users\Szymon\AppData\Local\Temp/ipykernel_61188/2236311633.py:5: UserWarning: This
pattern has match groups. To actually get the groups, use str.extract.
  synonymous=df['Annotation'].str.contains(r'^([A-Z])[0-9]+\1',regex=True).value_coun
ts()[True]
C:\Users\Szymon\AppData\Local\Temp/ipykernel_61188/2236311633.py:6: UserWarning: This
pattern has match groups. To actually get the groups, use str.extract.
  missense=df['Annotation'].str.contains(r'^([A-Z])[0-9]+[A-Z]',regex=True).value_cou
nts()[True]-synonymous
```

In [50]:

```
#https://link.springer.com/article/10.1186/s12864-015-1737-4#MOESM2
#Cell periphery-related proteins as major genomic targets behind the adaptive evolut
#It turns out that this data is not usefull, because it is hard to find information
df=pd.read_excel('Cell-periphery-related1.xlsx',sheet_name='ER Syn & Non-syn')
missense=df['Consequence'].str.contains('missense_variant').value_counts()[True]
synonymous=df['Consequence'].str.contains('synonymous_variant').value_counts()[True]
nonsense=df['Consequence'].str.contains('stop_gained').value_counts()[True]
statystyka_nonsense_essential_included(synonymous,nonsense,missense)
print(synonymous,nonsense,missense)
df=pd.read_excel('Cell-periphery-related2.xlsx',sheet_name='ISO12 Syn & Non-syn')
missense=df['Consequence'].str.contains('missense_variant').value_counts()[True]
synonymous=df['Consequence'].str.contains('synonymous_variant').value_counts()[True]
nonsense=df['Consequence'].str.contains('stop_gained').value_counts()[True]
statystyka_nonsense_essential_included(synonymous,nonsense,missense)
statystyka_nonsense_deleterious_excluded(synonymous,nonsense,missense)
print(synonymous,nonsense,missense)
synonymous=536-222
```

```
synonymous sides  5058099  synonymous mutations  536 ds 0.00010596866530291321
nonsense sides  1166436  nonsense mutations  7 dnonsense 6.0011865203063e-06
P-value nonsense mutation rate=synonymous mutation rate 3.568381926383667e-25
missense sides  17554023  missense mutations  400 dn 2.2786799356478e-05
P-value missense mutation rate=synonymous mutation rate 2.5941057657394517e-144
dNONSENSE/dS  0.05663170809174398
dN/dS  0.21503337133992914
536 7 400
synonymous sides  5058099  synonymous mutations  222 ds 4.389000689784838e-05
nonsense sides  1166436  nonsense mutations  15 dnonsense 1.2859685400656358e-05
P-value nonsense mutation rate=synonymous mutation rate 1.489318162019592e-06
missense sides  17554023  missense mutations  239 dn 1.3615112615495604e-05
P-value missense mutation rate=synonymous mutation rate 5.8512648395305795e-40
dNONSENSE/dS  0.29299802642060596
dN/dS  0.31020985362759324
The corrected dNONSENSE/dS calculations
synonymous sides  5058099  synonymous mutations  222 ds 4.389000689784838e-05
nonsense sides  233292  nonsense mutations  15 dnonsense 6.429710405843321e-05
P-value nonsense mutation rate=synonymous mutation rate 0.19994533791850522
dNONSENSE/dS  1.46495998982368
222 15 239
```

In [51]:

```
#The statistics calculated for the study:"Enhanced astaxanthin production in yeast v
#https://www.sciencedirect.com/science/article/pii/S1369703X20300346#fig0015
#I am not sure, what is exact number of nonsense and missense mutations, as figure i
#I wrote the e-mail to the authors and asked them,

statystyka_nonsense_essential_included(49,1,43)
statystyka_nonsense_deleterious_excluded(49,1,43)
statystyka_nonsense_essential_included(43,1,34)
statystyka_nonsense_deleterious_excluded(43,1,34)
statystyka_nonsense_essential_included(49,1,29)
statystyka_nonsense_deleterious_excluded(49,1,29)
```

```
synonymous sides  5058099  synonymous mutations  49 ds 9.687433954930498e-06
nonsense sides  1166436  nonsense mutations  1 dnonsense 8.573123600437573e-07
P-value nonsense mutation rate=synonymous mutation rate 0.004344179814402011
missense sides  17554023  missense mutations  43 dn 2.449580930821385e-06
P-value missense mutation rate=synonymous mutation rate 2.841670408984496e-12
dNONSENSE/dS  0.08849736308214222
dN/dS  0.2528616909511575
The corrected dNONSENSE/dS calculations
synonymous sides  5058099  synonymous mutations  49 ds 9.687433954930498e-06
nonsense sides  233292  nonsense mutations  1 dnonsense 4.286473603895547e-06
```

```
P-value nonsense mutation rate=synonymous mutation rate 0.6274822192197929
dNONSENSE/dS  0.4424777112120503
synonymous sides  5058099  synonymous mutations  43 ds 8.501217552285948e-06
nonsense sides  1166436  nonsense mutations  1 dnonsense 8.573123600437573e-07
P-value nonsense mutation rate=synonymous mutation rate 0.009163398290259667
missense sides  17554023  missense mutations  34 dn 1.9368779453006298e-06
P-value missense mutation rate=synonymous mutation rate 4.770547374933233e-12
dNONSENSE/dS  0.10084583234941787
dN/dS  0.22783535809877137
The corrected dNONSENSE/dS calculations
synonymous sides  5058099  synonymous mutations  43 ds 8.501217552285948e-06
nonsense sides  233292  nonsense mutations  1 dnonsense 4.286473603895547e-06
P-value nonsense mutation rate=synonymous mutation rate 0.7466609047055636
dNONSENSE/dS  0.504218787195127
synonymous sides  5058099  synonymous mutations  49 ds 9.687433954930498e-06
nonsense sides  1166436  nonsense mutations  1 dnonsense 8.573123600437573e-07
P-value nonsense mutation rate=synonymous mutation rate 0.004344179814402011
missense sides  17554023  missense mutations  29 dn 1.652042953344655e-06
P-value missense mutation rate=synonymous mutation rate 3.2479491471277763e-17
dNONSENSE/dS  0.08849736308214222
dN/dS  0.1705346287810132
The corrected dNONSENSE/dS calculations
synonymous sides  5058099  synonymous mutations  49 ds 9.687433954930498e-06
nonsense sides  233292  nonsense mutations  1 dnonsense 4.286473603895547e-06
P-value nonsense mutation rate=synonymous mutation rate 0.6274822192197929
dNONSENSE/dS  0.4424777112120503
```

In [52]:

```
#The statistics calculated for the study: "Phenotypic heterogeneity promotes adaptiv
#https://journals.plos.org/plosbiology/article?id=10.1371/journal.pbio.2000644
#6 nonsense, 6 synonymous, non-synonymous 30
#statystyka_nonsense_essential_excluded(6,6,30)
statystyka_nonsense_essential_included(6,6,30)
statystyka_nonsense_deleterious_excluded(6,6,30)
```

```
synonymous sides  5058099  synonymous mutations  6 ds 1.186216402644551e-06
nonsense sides  1166436  nonsense mutations  6 dnonsense 5.143874160262543e-06
P-value nonsense mutation rate=synonymous mutation rate 0.016164999710435748
missense sides  17554023  missense mutations  30 dn 1.70900995173585e-06
P-value missense mutation rate=synonymous mutation rate 0.5345622210282306
dNONSENSE/dS  4.336370791024968
dN/dS  1.440723587977525
The corrected dNONSENSE/dS calculations
synonymous sides  5058099  synonymous mutations  6 ds 1.186216402644551e-06
nonsense sides  233292  nonsense mutations  6 dnonsense 2.5718841623373285e-05
P-value nonsense mutation rate=synonymous mutation rate 2.7517429533316604e-12
dNONSENSE/dS  21.681407849390464
```

In [53]:

```
#The statistics calculated for the study:"Serial propagation in water-in-oil emulsio
#https://www.sciencedirect.com/science/article/pii/S1096717620301890

#synonymous=df['annotation'].str.contains(r'^([A-Z])[0-9]+\1',regex=True).value_coun
#missense=df['annotation'].str.contains(r'^([A-Z])[0-9]+[A-Z]',regex=True).value_cou
#nonsense=df['annotation'].str.contains(r'^([A-Z])[0-9]+\*',regex=True).value_counts
#statystyka_nonsense_essential_excluded(synonymous,nonsense,missense)
#statystyka_nonsense_essential_included(synonymous,nonsense,missense)
#print(synonymous,nonsense,missense)
df=pd.read_excel('Serial_propagation.xlsx',sheet_name='Without poly-nucleotide stret
synonymous=df['annotation'].str.contains(r'^([A-Z])[0-9]+\1',regex=True).value_count
missense=df['annotation'].str.contains(r'^([A-Z])[0-9]+[A-Z]',regex=True).value_coun
nonsense=df['annotation'].str.contains(r'^([A-Z])[0-9]+[\*]',regex=True).value_count
statystyka_nonsense_essential_included(synonymous,nonsense,missense)
statystyka_nonsense_deleterious_excluded(synonymous,nonsense,missense)
print(synonymous,nonsense,missense)
```

```python
df=pd.read_excel('Serial_propagation.xlsx',sheet_name='All mutations')
synonymous=df['annotation'].str.contains(r'^([A-Z])[0-9]+\1',regex=True).value_count
missense=df['annotation'].str.contains(r'^([A-Z])[0-9]+[A-Z]',regex=True).value_coun
nonsense=df['annotation'].str.contains(r'^([A-Z])[0-9]+[\*]',regex=True).value_count
statystyka_nonsense_essential_included(synonymous,nonsense,missense)
statystyka_nonsense_deleterious_excluded(synonymous,nonsense,missense)
print(synonymous,nonsense,missense)

#df1=df.dropna(subset=['EZ11','EZ21'])
#synonymous=df1['annotation'].str.contains(r'^([A-Z])[0-9]+\1',regex=True).value_cou
#missense=df1['annotation'].str.contains(r'^([A-Z])[0-9]+[A-Z]',regex=True).value_co
#nonsense=df['annotation'].str.contains(r'^([A-Z])[0-9]+\*',regex=True).value_counts
# jeśli bierze się tylko pod uwagę mutacje obecne w wielu eksperymentach
#nonsense=0
#statystyka_nonsense_essential_included(synonymous,nonsense,missense)
#print(synonymous,nonsense,missense)
#df1=df.fillna(0)
#df1[df1['EZ11'].str.contains('[0-9]')]
#df1=df.replace(np.nan, 0)
#df1['EZ21'].str.contains('^[1-9]')
```

```
C:\Users\Szymon\AppData\Local\Temp/ipykernel_61188/1888353191.py:11: UserWarning: Thi
s pattern has match groups. To actually get the groups, use str.extract.
  synonymous=df['annotation'].str.contains(r'^([A-Z])[0-9]+\1',regex=True).value_coun
ts()[True]
C:\Users\Szymon\AppData\Local\Temp/ipykernel_61188/1888353191.py:12: UserWarning: Thi
s pattern has match groups. To actually get the groups, use str.extract.
  missense=df['annotation'].str.contains(r'^([A-Z])[0-9]+[A-Z]',regex=True).value_cou
nts()[True]-synonymous
C:\Users\Szymon\AppData\Local\Temp/ipykernel_61188/1888353191.py:13: UserWarning: Thi
s pattern has match groups. To actually get the groups, use str.extract.
  nonsense=df['annotation'].str.contains(r'^([A-Z])[0-9]+[\*]',regex=True).value_coun
ts()[True]
C:\Users\Szymon\AppData\Local\Temp/ipykernel_61188/1888353191.py:18: UserWarning: Thi
s pattern has match groups. To actually get the groups, use str.extract.
  synonymous=df['annotation'].str.contains(r'^([A-Z])[0-9]+\1',regex=True).value_coun
ts()[True]
C:\Users\Szymon\AppData\Local\Temp/ipykernel_61188/1888353191.py:19: UserWarning: Thi
s pattern has match groups. To actually get the groups, use str.extract.
  missense=df['annotation'].str.contains(r'^([A-Z])[0-9]+[A-Z]',regex=True).value_cou
nts()[True]-synonymous
C:\Users\Szymon\AppData\Local\Temp/ipykernel_61188/1888353191.py:20: UserWarning: Thi
s pattern has match groups. To actually get the groups, use str.extract.
  nonsense=df['annotation'].str.contains(r'^([A-Z])[0-9]+[\*]',regex=True).value_coun
ts()[True]
synonymous sides  5058099  synonymous mutations  105 ds 2.075878704627964e-05
nonsense sides  1166436  nonsense mutations  13 dnonsense 1.1145060680568844e-05
P-value nonsense mutation rate=synonymous mutation rate 0.04218240667840562
missense sides  17554023  missense mutations  239 dn 1.3615112615495604e-05
P-value missense mutation rate=synonymous mutation rate 0.00036443101433017753
dNONSENSE/dS  0.5368840026983294
dN/dS  0.6558722619554829
The corrected dNONSENSE/dS calculations
synonymous sides  5058099  synonymous mutations  105 ds 2.075878704627964e-05
nonsense sides  233292  nonsense mutations  13 dnonsense 5.572415685064211e-05
P-value nonsense mutation rate=synonymous mutation rate 0.0010669368681586033
dNONSENSE/dS  2.684364781353105
105 13 239
synonymous sides  5058099  synonymous mutations  105 ds 2.075878704627964e-05
nonsense sides  1166436  nonsense mutations  13 dnonsense 1.1145060680568844e-05
P-value nonsense mutation rate=synonymous mutation rate 0.04218240667840562
missense sides  17554023  missense mutations  239 dn 1.3615112615495604e-05
P-value missense mutation rate=synonymous mutation rate 0.00036443101433017753
dNONSENSE/dS  0.5368840026983294
```

```
dN/dS  0.6558722619554829
The corrected dNONSENSE/dS calculations
synonymous sides  5058099  synonymous mutations  105 ds 2.075878704627964e-05
nonsense sides  233292  nonsense mutations  13 dnonsense 5.572415685064211e-05
P-value nonsense mutation rate=synonymous mutation rate 0.0010669368681586033
dNONSENSE/dS  2.684364781353105
105 13 239
```

In [54]:

```
#The calculation of statistics for the study: "Chance and necessity in the pleiotrop
#https://www.nature.com/articles/s41559-020-1128-3
#https://static-content.springer.com/esm/art%3A10.1038%2Fs41559-020-1128-3/MediaObje
#synonymous 300
#non-synonymous 1096
#nonsense 74
#FS 96
print(1096-74-96)
statystyka_nonsense_essential_included(300,74,926)
statystyka_nonsense_deleterious_excluded(300,74,926)
```

```
926
synonymous sides  5058099  synonymous mutations  300 ds 5.9310820132227545e-05
nonsense sides  1166436  nonsense mutations  74 dnonsense 6.344111464323804e-05
P-value nonsense mutation rate=synonymous mutation rate 0.6509156576930364
missense sides  17554023  missense mutations  926 dn 5.275144051024657e-05
P-value missense mutation rate=synonymous mutation rate 0.08346902397214849
dNONSENSE/dS  1.0696381284528256
dN/dS  0.8894066949781255
The corrected dNONSENSE/dS calculations
synonymous sides  5058099  synonymous mutations  300 ds 5.9310820132227545e-05
nonsense sides  233292  nonsense mutations  74 dnonsense 0.0003171990466882705
P-value nonsense mutation rate=synonymous mutation rate 9.554477224929296e-47
dNONSENSE/dS  5.348080602849647
```

In [55]:

```
#The calculation of statistics for the study : "Genomic, transcriptomic and physiolo
#https://onlinelibrary.wiley.com/doi/full/10.1002/yea.3514
#"The mutant had three stop-loss, 61 nonsynonymous, three synonymous and 12 intergen
statystyka_nonsense_essential_included(3,0,61)
statystyka_nonsense_deleterious_excluded(3,0,61)
```

```
synonymous sides  5058099  synonymous mutations  3 ds 5.931082013222755e-07
nonsense sides  1166436  nonsense mutations  0 dnonsense 0.0
P-value nonsense mutation rate=synonymous mutation rate 0.9267010472100584
missense sides  17554023  missense mutations  61 dn 3.474986901862895e-06
P-value missense mutation rate=synonymous mutation rate 0.0011767679898450174
dNONSENSE/dS  0.0
dN/dS  5.858942591108602
The corrected dNONSENSE/dS calculations
synonymous sides  5058099  synonymous mutations  3 ds 5.931082013222755e-07
nonsense sides  233292  nonsense mutations  0 dnonsense 0.0
P-value nonsense mutation rate=synonymous mutation rate 1.0
dNONSENSE/dS  0.0
```

In [56]:

```
#The calculation of statistics for the study: "Reduced sensitivity of lager brewing
#https://www.sciencedirect.com/science/article/pii/S0740002022000569#appsec1
#The calculation were done using the Pandas package.
df=pd.read_excel('Reduced_evolution.xlsx')
missense=df['Variant type'].str.contains('missense_variant').value_counts()[True]
synonymous=df['Variant type'].str.contains('synonymous_variant').value_counts()[True
nonsense=df['Variant type'].str.contains('stop_gained').value_counts()[True]
statystyka_nonsense_essential_included(synonymous,nonsense,missense)
print(synonymous,nonsense,missense)
statystyka_nonsense_deleterious_excluded(synonymous,nonsense,missense)
```

```
#df1=df.replace(np.nan, 0)
#df2=df1[(df1['TT02-10S']=='0/1')&(df1['TT01-20T']=='0/0')&(df1['TT02-30S']=='0/0')&
#missense=df2['Variant type'].str.contains('missense_variant').value_counts()[True]
#synonymous=df2['Variant type'].str.contains('synonymous_variant').value_counts()[Tr
#nonsense=df2['Variant type'].str.contains('stop_gained').value_counts()[True]
#statystyka_nonsense_essential_excluded(synonymous,nonsense,missense)
#statystyka_nonsense_essential_included(synonymous,nonsense,missense)
#print(synonymous,nonsense,missense)
#df2
#df2=df1[(df1['TT02-10S']=='0/0')&(df1['TT01-20T']=='0/0')&(df1['TT02-30S']=='0/1')&
#missense=df2['Variant type'].str.contains('missense_variant').value_counts()[True]
#synonymous=df2['Variant type'].str.contains('synonymous_variant').value_counts()[Tr
#nonsense=df2['Variant type'].str.contains('stop_gained').value_counts()[True]
#statystyka_nonsense_essential_excluded(synonymous,nonsense,missense)
#statystyka_nonsense_essential_included(synonymous,nonsense,missense)
#print(synonymous,nonsense,missense)
#df2
#df2
#df2=df1[(df1['TT02-10S']=='0/0')&(df1['TT01-20T']=='0/0')&(df1['TT02-30S']=='0/0')&
#df2
#missense=df2['Variant type'].str.contains('missense_variant').value_counts()[True]
#synonymous=df2['Variant type'].str.contains('synonymous_variant').value_counts()[Tr
#nonsense=df2['Variant type'].str.contains('stop_gained').value_counts()[True]
#statystyka_nonsense_essential_excluded(synonymous,nonsense,missense)
#statystyka_nonsense_essential_included(synonymous,nonsense,missense)
#print(synonymous,nonsense,missense)
```

```
synonymous sides  5058099  synonymous mutations  53 ds 1.0478244890026865e-05
nonsense sides  1166436  nonsense mutations  4 dnonsense 3.429249440175029e-06
P-value nonsense mutation rate=synonymous mutation rate 0.03589381728877474
missense sides  17554023  missense mutations  58 dn 3.30408590668931e-06
P-value missense mutation rate=synonymous mutation rate 2.929067606800601e-10
dNONSENSE/dS  0.3272732672471675
dN/dS  0.3153281815196093
53 4 58
The corrected dNONSENSE/dS calculations
synonymous sides  5058099  synonymous mutations  53 ds 1.0478244890026865e-05
nonsense sides  233292  nonsense mutations  4 dnonsense 1.714589441558219e-05
P-value nonsense mutation rate=synonymous mutation rate 0.5242881375976767
dNONSENSE/dS  1.6363326678785257
```

In [57]:

```
#The calculation of statistics for the study:"Laboratory evolution reveals regulator
#https://www.sciencedirect.com/science/article/pii/S1096717617304032#s0120
#The calculations were performed using the Pandas package.

df=pd.read_excel('Laboratory_evolution_reveals.xlsx')
missense=df['AA Mutation'].str.contains(r'^([A-Z])[0-9]+[A-Z]').value_counts()[True]
nonsense=df['AA Mutation'].str.contains(r'^[0-9]+\*').value_counts()[True]
synonymous=df['AA Mutation'].str.contains('SILENT').value_counts()[True]
#synonymous=df['Variant type'].str.contains('synonymous_variant').value_counts()[Tru
#nonsense=df['Variant type'].str.contains('stop_gained').value_counts()[True]
#statystyka_nonsense_essential_excluded(synonymous,nonsense,missense)
statystyka_nonsense_essential_included(synonymous,nonsense,missense)
statystyka_nonsense_deleterious_excluded(synonymous,nonsense,missense)
synonymous
```

```
synonymous sides  5058099  synonymous mutations  2 ds 3.954054675481836e-07
nonsense sides  1166436  nonsense mutations  2 dnonsense 1.7146247200875146e-06
P-value nonsense mutation rate=synonymous mutation rate 0.33628814419236197
missense sides  17554023  missense mutations  27 dn 1.538108956562265e-06
P-value missense mutation rate=synonymous mutation rate 0.07562271697649459
dNONSENSE/dS  4.336370791024969
dN/dS  3.8899536875393177
The corrected dNONSENSE/dS calculations
```

```
synonymous sides  5058099  synonymous mutations  2 ds 3.954054675481836e-07
nonsense sides  233292  nonsense mutations  2 dnonsense 8.572947207791094e-06
P-value nonsense mutation rate=synonymous mutation rate 0.001265099007275029
dNONSENSE/dS  21.681407849390464
```

```
C:\Users\Szymon\AppData\Local\Temp/ipykernel_61188/498813084.py:6: UserWarning: This
pattern has match groups. To actually get the groups, use str.extract.
  missense=df['AA Mutation'].str.contains(r'^([A-Z])[0-9]+[A-Z]').value_counts()[Tru
e]
```

Out[57]:

```
2
```

In [58]:

```python
#The calculations of statistics for the study: "Altered sterol composition renders y
#https://www.science.org/doi/full/10.1126/science.1258137
statystyka_nonsense_essential_included(3,6,12)
statystyka_nonsense_deleterious_excluded(3,6,12)
```

```
synonymous sides  5058099  synonymous mutations  3 ds 5.931082013222755e-07
nonsense sides  1166436  nonsense mutations  6 dnonsense 5.143874160262543e-06
P-value nonsense mutation rate=synonymous mutation rate 0.0011241205718903024
missense sides  17554023  missense mutations  12 dn 6.8360398069434e-07
P-value missense mutation rate=synonymous mutation rate 1.0
dNONSENSE/dS  8.672741582049936
dN/dS  1.15257887038202
The corrected dNONSENSE/dS calculations
synonymous sides  5058099  synonymous mutations  3 ds 5.931082013222755e-07
nonsense sides  233292  nonsense mutations  6 dnonsense 2.5718841623373285e-05
P-value nonsense mutation rate=synonymous mutation rate 1.1717351896971425e-16
dNONSENSE/dS  43.36281569878093
```

In [59]:

```python
#The calculations for the study: "Genome-scale analyses of butanol tolerance in Sacc
#https://biotechnologyforbiofuels.biomedcentral.com/articles/10.1186/1754-6834-6-48#
#I couldn't find number of synonymous mutations
statystyka_nonsense_essential_included(0,3,6)
statystyka_nonsense_deleterious_excluded(0,3,6)
```

```
synonymous sides  5058099  synonymous mutations  0 ds 0.0
nonsense sides  1166436  nonsense mutations  3 dnonsense 2.5719370801312716e-06
P-value nonsense mutation rate=synonymous mutation rate 0.004143187245118398
missense sides  17554023  missense mutations  6 dn 3.4180199034717e-07
P-value missense mutation rate=synonymous mutation rate 0.40935750139635696
dNONSENSE/dS  error ds=0
dN/dS  error ds=0
The corrected dNONSENSE/dS calculations
synonymous sides  5058099  synonymous mutations  0 ds 0.0
nonsense sides  233292  nonsense mutations  3 dnonsense 1.2859420811686642e-05
P-value nonsense mutation rate=synonymous mutation rate 2.760812160641767e-11
dNONSENSE/dS  error ds=0
```

In [ ]:

```python

```

In [60]:

```python
#The calculation of statistic for the study: "Deconstructing the genetic basis of sp
#https://biotechnologyforbiofuels.biomedcentral.com/articles/10.1186/s13068-015-0241
#Deconstructing the genetic basis of spent sulphite liquor tolerance using deep sequ
statystyka_nonsense_essential_included(2,0,10)
statystyka_nonsense_deleterious_excluded(2,0,10)
```

```
synonymous sides  5058099  synonymous mutations  2 ds 3.954054675481836e-07
nonsense sides  1166436  nonsense mutations  0 dnonsense 0.0
P-value nonsense mutation rate=synonymous mutation rate 1.0
missense sides  17554023  missense mutations  10 dn 5.6966998391195e-07
P-value missense mutation rate=synonymous mutation rate 0.8984219710036399
```

```
dNONSENSE/dS  0.0
dN/dS  1.440723587977525
The corrected dNONSENSE/dS calculations
synonymous sides  5058099  synonymous mutations  2 ds 3.954054675481836e-07
nonsense sides  233292  nonsense mutations  0 dnonsense 0.0
P-value nonsense mutation rate=synonymous mutation rate 1.0
dNONSENSE/dS  0.0
```

In [61]:

```
#The calculations of statistics for the study:"Adaptive Roles of SSY1 and SIR3 Durin
#https://academic.oup.com/g3journal/article/7/6/1899/6029837
#See Figure 4
statystyka_nonsense_essential_included(0,6,5)
statystyka_nonsense_deleterious_excluded(0,6,5)
```

```
synonymous sides  5058099  synonymous mutations  0 ds 0.0
nonsense sides  1166436  nonsense mutations  6 dnonsense 5.143874160262543e-06
P-value nonsense mutation rate=synonymous mutation rate 4.701043277841164e-06
missense sides  17554023  missense mutations  5 dn 2.84834991955975e-07
P-value missense mutation rate=synonymous mutation rate 0.5068764811321911
dNONSENSE/dS  error ds=0
dN/dS  error ds=0
The corrected dNONSENSE/dS calculations
synonymous sides  5058099  synonymous mutations  0 ds 0.0
nonsense sides  233292  nonsense mutations  6 dnonsense 2.5718841623373285e-05
P-value nonsense mutation rate=synonymous mutation rate 2.204351162143243e-25
dNONSENSE/dS  error ds=0
```

In [ ]:

```
#https://academic.oup.com/g3journal/article/7/4/1251/6031812?login=true
#Genome-Wide Screen Reveals sec21 Mutants of Saccharomyces cerevisiae Are Methotrexa
#Whole mitochondrial DNA is lost
```

In [62]:

```
#The calculations of statistics for the study: "Directed Evolution Reveals Unexpecte
#https://journals.plos.org/plosgenetics/article?id=10.1371/journal.pgen.1006372
#There is no information about synonymous mutations, see table 2.
statystyka_nonsense_essential_included(0,1,4)
statystyka_nonsense_deleterious_excluded(0,1,4)
```

```
synonymous sides  5058099  synonymous mutations  0 ds 0.0
nonsense sides  1166436  nonsense mutations  1 dnonsense 8.573123600437573e-07
P-value nonsense mutation rate=synonymous mutation rate 0.4230800402270193
missense sides  17554023  missense mutations  4 dn 2.2786799356477998e-07
P-value missense mutation rate=synonymous mutation rate 0.6357467590259751
dNONSENSE/dS  error ds=0
dN/dS  error ds=0
The corrected dNONSENSE/dS calculations
synonymous sides  5058099  synonymous mutations  0 ds 0.0
nonsense sides  233292  nonsense mutations  1 dnonsense 4.286473603895547e-06
P-value nonsense mutation rate=synonymous mutation rate 0.02636591897111694
dNONSENSE/dS  error ds=0
```

In [63]:

```
#The calculation of statistics for the study:"Rational and Evolutionary Engineering 
#https://journals.plos.org/plosone/article?id=10.1371/journal.pone.0057048
#See the table S1
#10 missense,0 nonsense, 18 synonymous
statystyka_nonsense_essential_included(18,0,10)
statystyka_nonsense_deleterious_excluded(18,0,10)
```

```
synonymous sides  5058099  synonymous mutations  18 ds 3.5586492079336526e-06
nonsense sides  1166436  nonsense mutations  0 dnonsense 0.0
P-value nonsense mutation rate=synonymous mutation rate 0.08267412143367983
missense sides  17554023  missense mutations  10 dn 5.6966998391195e-07
```

```
P-value missense mutation rate=synonymous mutation rate 3.47150907923672e-07
dNONSENSE/dS  0.0
dN/dS  0.16008039866416945
The corrected dNONSENSE/dS calculations
synonymous sides  5058099  synonymous mutations  18 ds 3.5586492079336526e-06
nonsense sides  233292  nonsense mutations  0 dnonsense 0.0
P-value nonsense mutation rate=synonymous mutation rate 0.7360491064275976
dNONSENSE/dS  0.0
```

In [ ]:

```
#https://link.springer.com/article/10.1186/s12864-015-1755-2
#Evolutionary engineering of a wine yeast strain revealed a key role of inositol and
#I couldn't find information about synonymous mutations
#The whole genome of evolved mutant P5-EM was sequenced and compared with that of pa
#nonsense mutations also could be not mentioned
```

In [ ]:

```

```

In [ ]:

```
#https://onlinelibrary.wiley.com/doi/full/10.1111/j.1420-9101.2011.02249.x
#Adaptation of Saccharomyces cerevisiae to saline stress through laboratory evolutio
#There is only one missense mutation. However, this is experiment of the Andreas Wagi
```

In [ ]:

```

```

In [64]:

```
#The calculations of statistics for the study:"Heterozygote Advantage Is a Common Ou
#https://academic.oup.com/genetics/article/203/3/1401/6065806?login=true
#See table. I used only non-redundant mutations:synonymous 1, nonsense 1, 5 missense
statystyka_nonsense_essential_included(1,1,5)
statystyka_nonsense_deleterious_excluded(1,1,5)
```

```
synonymous sides  5058099  synonymous mutations  1 ds 1.977027337740918e-07
nonsense sides  1166436  nonsense mutations  1 dnonsense 8.573123600437573e-07
P-value nonsense mutation rate=synonymous mutation rate 0.8205081794951997
missense sides  17554023  missense mutations  5 dn 2.84834991955975e-07
P-value missense mutation rate=synonymous mutation rate 1.0
dNONSENSE/dS  4.336370791024969
dN/dS  1.440723587977525
The corrected dNONSENSE/dS calculations
synonymous sides  5058099  synonymous mutations  1 ds 1.977027337740918e-07
nonsense sides  233292  nonsense mutations  1 dnonsense 4.286473603895547e-06
P-value nonsense mutation rate=synonymous mutation rate 0.1560539506567918
dNONSENSE/dS  21.681407849390464
```

In [67]:

```
#The calculations of statistics for the study:"Engineering a Balanced Acetyl Coenzym
#https://pubs.acs.org/doi/full/10.1021/acs.jafc.2c00531
#https://pubs.acs.org/doi/10.1021/acs.jafc.2c00531?goto=supporting-info
#They observed four mutations, two indels, two snps, only one nonsense, zero missens
#statystyka_nonsense_essential_included(0,1,0)
statystyka_nonsense_deleterious_excluded(0,1,0)
statystyka_nonsense_essential_included(0,1,0)
```

```
The corrected dNONSENSE/dS calculations
synonymous sides  5058099  synonymous mutations  0 ds 0.0
nonsense sides  233292  nonsense mutations  1 dnonsense 4.286473603895547e-06
P-value nonsense mutation rate=synonymous mutation rate 0.02636591897111694
dNONSENSE/dS  error ds=0
synonymous sides  5058099  synonymous mutations  0 ds 0.0
nonsense sides  1166436  nonsense mutations  1 dnonsense 8.573123600437573e-07
```

```
P-value nonsense mutation rate=synonymous mutation rate 0.4230800402270193
missense sides  17554023  missense mutations  0 dn 0.0
```

```
---------------------------------------------------------------------------
ValueError                                Traceback (most recent call last)
~\AppData\Local\Temp/ipykernel_61188/4294288310.py in <module>
      5 #statystyka_nonsense_essential_included(0,1,0)
      6 statystyka_nonsense_deleterious_excluded(0,1,0)
----> 7 statystyka_nonsense_essential_included(0,1,0)

~\AppData\Local\Temp/ipykernel_61188/4072840567.py in statystyka_nonsense_essential_i
ncluded(synony, nonsen, missense)
     21         print("missense sides ",tablica_wynikow[2], " missense mutations ",mis
sense,"dn",dn_rate)
     22         tab_data = [[synony,tablica_wynikow[0]], [missense,tablica_wynikow
[2]]]
---> 23         pvalue=chi2_contingency(tab_data)[1]
     24         print("P-value missense mutation rate=synonymous mutation rate",pvalu
e)
     25         print("dNONSENSE/dS ",dnonsense_to_ds)

~\anaconda3\lib\site-packages\scipy\stats\contingency.py in chi2_contingency(observe
d, correction, lambda_)
    274         # the exception message.
    275         zeropos = list(zip(*np.nonzero(expected == 0)))[0]
--> 276         raise ValueError("The internally computed table of expected "
    277                          "frequencies has a zero element at %s." % (zeropo
s,))
    278

ValueError: The internally computed table of expected frequencies has a zero element
at (0, 0).
```

In [68]:

```
#The calculation of statistics for the study:"A new laboratory evolution approach to
#https://biotechnologyforbiofuels.biomedcentral.com/articles/10.1186/s13068-016-0583
#The calculations were made using the Pandas package.
#A new laboratory evolution approach to select for constitutive acetic acid toleranc
#A_new_laboratory_evolution_approach file
df=pd.read_excel("A_new_laboratory_evolution_approach.xlsx")
synonymous=df['Aminoacid change'].str.contains('None').value_counts()[True]-4 #4 non
missense=df['Aminoacid change'].str.contains('[0-9][A-Z]').value_counts()[True]
nonsense=df['Aminoacid change'].str.contains('[0-9]\*').value_counts()[True]
print(synonymous,missense,nonsense)
statystyka_nonsense_essential_included(synonymous,nonsense,missense)
statystyka_nonsense_deleterious_excluded(synonymous,nonsense,missense)
```

```
9 20 3
synonymous sides  5058099  synonymous mutations  9 ds 1.7793246039668263e-06
nonsense sides  1166436  nonsense mutations  3 dnonsense 2.5719370801312716e-06
P-value nonsense mutation rate=synonymous mutation rate 0.8525333470365942
missense sides  17554023  missense mutations  20 dn 1.1393399678239e-06
P-value missense mutation rate=synonymous mutation rate 0.3697069859905824
dNONSENSE/dS  1.445456930341656
dN/dS  0.6403215946566778
The corrected dNONSENSE/dS calculations
synonymous sides  5058099  synonymous mutations  9 ds 1.7793246039668263e-06
nonsense sides  233292  nonsense mutations  3 dnonsense 1.2859420811686642e-05
P-value nonsense mutation rate=synonymous mutation rate 0.005581011821056402
dNONSENSE/dS  7.227135949796821
```

In [69]:

```
#The calculation of the statistics for the study:"Evolutionary engineering reveals d
#https://www.sciencedirect.com/science/article/pii/S1096717616301756
#3 nonsense mutations, 11 non-synonymous mutations, 3 synonymous mutations a
statystyka_nonsense_essential_included(3,3,11)
```

```
statystyka_nonsense_deleterious_excluded(3,3,11)
#The Glu+LA strains presented a different set of mutations with a total of 11 SNVs i
statystyka_nonsense_essential_included(0,2,6)
statystyka_nonsense_deleterious_excluded(0,2,6)
#In the genomes of the five strains from the Raf+LA cultures, we detected a total of
statystyka_nonsense_essential_included(0,1,11)
statystyka_nonsense_deleterious_excluded(0,1,11)
```

```
synonymous sides  5058099  synonymous mutations  3 ds 5.931082013222755e-07
nonsense sides  1166436  nonsense mutations  3 dnonsense 2.5719370801312716e-06
P-value nonsense mutation rate=synonymous mutation rate 0.15010297677358228
missense sides  17554023  missense mutations  11 dn 6.26636982303145e-07
P-value missense mutation rate=synonymous mutation rate 1.0
dNONSENSE/dS  4.336370791024968
dN/dS  1.0565306311835183
The corrected dNONSENSE/dS calculations
synonymous sides  5058099  synonymous mutations  3 ds 5.931082013222755e-07
nonsense sides  233292  nonsense mutations  3 dnonsense 1.2859420811686642e-05
P-value nonsense mutation rate=synonymous mutation rate 8.770712870884365e-06
dNONSENSE/dS  21.681407849390464
synonymous sides  5058099  synonymous mutations  0 ds 0.0
nonsense sides  1166436  nonsense mutations  2 dnonsense 1.7146247200875146e-06
P-value nonsense mutation rate=synonymous mutation rate 0.041457000496585586
missense sides  17554023  missense mutations  6 dn 3.4180199034717e-07
P-value missense mutation rate=synonymous mutation rate 0.40935750139635696
dNONSENSE/dS  error ds=0
dN/dS  error ds=0
The corrected dNONSENSE/dS calculations
synonymous sides  5058099  synonymous mutations  0 ds 0.0
nonsense sides  233292  nonsense mutations  2 dnonsense 8.572947207791094e-06
P-value nonsense mutation rate=synonymous mutation rate 1.1571721901605702e-06
dNONSENSE/dS  error ds=0
synonymous sides  5058099  synonymous mutations  0 ds 0.0
nonsense sides  1166436  nonsense mutations  1 dnonsense 8.573123600437573e-07
P-value nonsense mutation rate=synonymous mutation rate 0.4230800402270193
missense sides  17554023  missense mutations  11 dn 6.26636982303145e-07
P-value missense mutation rate=synonymous mutation rate 0.15602612966042279
dNONSENSE/dS  error ds=0
dN/dS  error ds=0
The corrected dNONSENSE/dS calculations
synonymous sides  5058099  synonymous mutations  0 ds 0.0
nonsense sides  233292  nonsense mutations  1 dnonsense 4.286473603895547e-06
P-value nonsense mutation rate=synonymous mutation rate 0.02636591897111694
dNONSENSE/dS  error ds=0
```

In [70]:

```
#The calculation of statistics for the study:"Complete genome sequence and analysis
#https://academic.oup.com/dnaresearch/article/25/3/297/4838783
#See table 2
#Synonymous      23,717  23,350  367     75      21      54
# Nonsynonymous          12,380  12,121  259     72      26      46
# Frameshift     274     243     31      6       2       4
# Nonframeshift          1,996   1,748   248     55      20      35
# Stop gain or loss      89      82      7       6       4       2nese rice wine prod
statystyka_nonsense_essential_included(23717,89,12380)
statystyka_nonsense_deleterious_excluded(23717,89,12380)
```

```
synonymous sides  5058099  synonymous mutations  23717 ds 0.004688915736920135
nonsense sides  1166436  nonsense mutations  89 dnonsense 7.63008000438944e-05
P-value nonsense mutation rate=synonymous mutation rate 0.0
missense sides  17554023  missense mutations  12380 dn 0.0007052514400829941
P-value missense mutation rate=synonymous mutation rate 0.0
dNONSENSE/dS  0.01627258929886673
dN/dS  0.15040821367931662
```

```
The corrected dNONSENSE/dS calculations
synonymous sides  5058099  synonymous mutations  23717 ds 0.004688915736920135
nonsense sides  233292  nonsense mutations  89 dnonsense 0.0003814961507467037
P-value nonsense mutation rate=synonymous mutation rate 1.0897749317377075e-201
dNONSENSE/dS  0.08136127244574572
```

In [79]:

```
#The calculation of statistics for the study:"Genome sequencing and genetic breeding
#https://bmcgenomics.biomedcentral.com/articles/10.1186/1471-2164-13-479
# 142 ORFs had in-frame stop codons, 129 ORF were affected by frame shifts, and 27 O
#A total of 39,098 SNPs were found in the ORFs and 38.7% of them resulted in non-syn
print(39098*0.387)
print(39098*(1-0.387))
statystyka_nonsense_essential_included(23967,142,15131)
statystyka_nonsense_deleterious_excluded(23967,142,15131)
```

```
15130.926000000001
23967.074
synonymous sides  5058099  synonymous mutations  23967 ds 0.004738341420363658
nonsense sides  1166436  nonsense mutations  142 dnonsense 0.00012173835512621353
P-value nonsense mutation rate=synonymous mutation rate 0.0
missense sides  17554023  missense mutations  15131 dn 0.0008619676526571715
P-value missense mutation rate=synonymous mutation rate 0.0
dNONSENSE/dS  0.02569218727106211
dN/dS  0.1819133692968493
The corrected dNONSENSE/dS calculations
synonymous sides  5058099  synonymous mutations  23967 ds 0.004738341420363658
nonsense sides  233292  nonsense mutations  142 dnonsense 0.0006086792517531677
P-value nonsense mutation rate=synonymous mutation rate 3.3580463063976136e-183
dNONSENSE/dS  0.12845829326212901
```

In [77]:

```
#The calculation of statistics for the study:" Catalog of Neutral and Deleterious Po
#https://journals.plos.org/plosgenetics/article?id=10.1371/journal.pgen.1000183
#We used the Pandas package for calculations and table 1.
#Table 1
#https://journals.plos.org/plosgenetics/article?id=10.1371/journal.pgen.1000183
#Table S3.
#Strain-specific variation.
#12 943 non synonymous
#26 257 synonymous
df=pd.read_excel('A_katalog.xlsx',sheet_name='Dataset 4')
#df=pd.read_excel("A_new_laboratory_evolution_approach.xlsx")
nonsense=df['Unnamed: 15'].str.contains('->Stop').value_counts()[True]
missense=12943-nonsense
synonymous=26257
print(synonymous,missense,nonsense)
statystyka_nonsense_essential_included(synonymous,nonsense,missense)
statystyka_nonsense_deleterious_excluded(synonymous,nonsense,missense)
```

```
26257 12861 82
synonymous sides  5058099  synonymous mutations  26257 ds 0.005191080680706329
nonsense sides  1166436  nonsense mutations  82 dnonsense 7.029961352358809e-05
P-value nonsense mutation rate=synonymous mutation rate 0.0
missense sides  17554023  missense mutations  12861 dn 0.0007326525663091588
P-value missense mutation rate=synonymous mutation rate 0.0
dNONSENSE/dS  0.013542385073087077
dN/dS  0.14113680972676962
The corrected dNONSENSE/dS calculations
synonymous sides  5058099  synonymous mutations  26257 ds 0.005191080680706329
nonsense sides  233292  nonsense mutations  82 dnonsense 0.00035149083551943487
P-value nonsense mutation rate=synonymous mutation rate 7.523453255096187e-230
dNONSENSE/dS  0.0677105321876078
```

In [16]:

In [80]:

```
#Calculations of statistics for non-mutators evolving in continuous culture (see fil
statystyka_nonsense_essential_included(7,8,28)
statystyka_nonsense_deleterious_excluded(7,8,28)
#Calculations of statistics for mutators evolving in continuous culture  (see file p
statystyka_nonsense_essential_included(38,10,106)
statystyka_nonsense_deleterious_excluded(38,10,106)
```

```
synonymous sides  5058099  synonymous mutations  7 ds 1.3839191364186426e-06
nonsense sides  1166436  nonsense mutations  8 dnonsense 6.858498880350058e-06
P-value nonsense mutation rate=synonymous mutation rate 0.0019183070431539084
missense sides  17554023  missense mutations  28 dn 1.5950759549534599e-06
P-value missense mutation rate=synonymous mutation rate 0.8937923373333485
dNONSENSE/dS  4.955852332599965
dN/dS  1.1525788703820201
The corrected dNONSENSE/dS calculations
synonymous sides  5058099  synonymous mutations  7 ds 1.3839191364186426e-06
nonsense sides  233292  nonsense mutations  8 dnonsense 3.429178883116438e-05
P-value nonsense mutation rate=synonymous mutation rate 7.908144866896906e-18
dNONSENSE/dS  24.778751827874817
synonymous sides  5058099  synonymous mutations  38 ds 7.512703883415489e-06
nonsense sides  1166436  nonsense mutations  10 dnonsense 8.573123600437572e-06
P-value nonsense mutation rate=synonymous mutation rate 0.8517914309222397
missense sides  17554023  missense mutations  106 dn 6.03850182946667e-06
P-value missense mutation rate=synonymous mutation rate 0.29023533499642395
dNONSENSE/dS  1.1411502081644653
dN/dS  0.803772106976935
The corrected dNONSENSE/dS calculations
synonymous sides  5058099  synonymous mutations  38 ds 7.512703883415489e-06
nonsense sides  233292  nonsense mutations  10 dnonsense 4.2864736038955474e-05
P-value nonsense mutation rate=synonymous mutation rate 2.0888379997859134e-07
dNONSENSE/dS  5.705633644576437
```
